# Association between pregnancy-related complications and development of type 2 diabetes and hypertension in women: an umbrella review

**DOI:** 10.1186/s12916-024-03284-4

**Published:** 2024-02-14

**Authors:** Steven Wambua, Megha Singh, Kelvin Okoth, Kym I. E. Snell, Richard D. Riley, Christopher Yau, Shakila Thangaratinam, Krishnarajah Nirantharakumar, Francesca L. Crowe, on behalf of the MuM-PreDiCT Group

**Affiliations:** 1 Institute of Applied Health Research, College of Medical and Dental Sciences, University of Birmingham, Edgbaston, Birmingham, UK; 2 Big Data Institute, University of Oxford, Li Ka Shing Centre for Health Information and Discovery, Old Road Campus, Oxford, OX3 7LF UK; 3 Nuffield Department of Women’s & Reproductive Health, University of Oxford, Level 3 Women’s Centre, John Radcliffe Hospital, Oxford, OX3 9DU UK; 4 Health Data Research, London, UK; 5 WHO Collaborating Centre for Global Women’s Health, Institute of Metabolism and Systems Research, University of Birmingham, Birmingham, UK; 6 Department of Obstetrics and Gynaecology, Birmingham Women’s and Children’s NHS Foundation Trust, Birmingham, UK

**Keywords:** Pregnancy complications, Type 2 diabetes, Hypertension, Obstetrics and gynaecology, Umbrella review

## Abstract

**Background:**

Despite many systematic reviews and meta-analyses examining the associations of pregnancy complications with risk of type 2 diabetes mellitus (T2DM) and hypertension, previous umbrella reviews have only examined a single pregnancy complication. Here we have synthesised evidence from systematic reviews and meta-analyses on the associations of a wide range of pregnancy-related complications with risk of developing T2DM and hypertension.

**Methods:**

Medline, Embase and Cochrane Database of Systematic Reviews were searched from inception until 26 September 2022 for systematic reviews and meta-analysis examining the association between pregnancy complications and risk of T2DM and hypertension. Screening of articles, data extraction and quality appraisal (AMSTAR2) were conducted independently by two reviewers using Covidence software. Data were extracted for studies that examined the risk of T2DM and hypertension in pregnant women with the pregnancy complication compared to pregnant women without the pregnancy complication. Summary estimates of each review were presented using tables, forest plots and narrative synthesis and reported following Preferred Reporting Items for Overviews of Reviews (PRIOR) guidelines.

**Results:**

Ten systematic reviews were included. Two pregnancy complications were identified. *Gestational diabetes mellitus (GDM)*: One review showed GDM was associated with a 10-fold higher risk of T2DM at least 1 year after pregnancy (relative risk (RR) 9.51 (95% confidence interval (CI) 7.14 to 12.67) and although the association differed by ethnicity (*white: RR* 16.28 (95% CI 15.01 to 17.66), *non-white: RR* 10.38 (95% CI 4.61 to 23.39), *mixed: RR* 8.31 (95% CI 5.44 to 12.69)), the between subgroups difference were not statistically significant at 5% significance level. Another review showed GDM was associated with higher mean blood pressure at least 3 months postpartum (*mean difference in systolic blood pressure*: 2.57 (95% CI 1.74 to 3.40) mmHg and *mean difference in diastolic blood pressure:* 1.89 (95% CI 1.32 to 2.46) mmHg). *Hypertensive disorders of pregnancy (HDP)*: Three reviews showed women with a history of HDP were 3 to 6 times more likely to develop hypertension at least 6 weeks after pregnancy compared to women without HDP (meta-analysis with largest number of studies: odds ratio (OR) 4.33 (3.51 to 5.33)) and one review reported a higher rate of T2DM after HDP (hazard ratio (HR) 2.24 (1.95 to 2.58)) at least a year after pregnancy. One of the three reviews and five other reviews reported women with a history of preeclampsia were 3 to 7 times more likely to develop hypertension at least 6 weeks postpartum (meta-analysis with the largest number of studies: OR 3.90 (3.16 to 4.82) with one of these reviews reporting the association was greatest in women from Asia (*Asia: OR* 7.54 (95% CI 2.49 to 22.81), *Europe: OR* 2.19 (95% CI 0.30 to 16.02), *North and South America: OR* 3.32 (95% CI 1.26 to 8.74)).

**Conclusions:**

GDM and HDP are associated with a greater risk of developing T2DM and hypertension. Common confounders adjusted for across the included studies in the reviews were maternal age, body mass index (BMI), socioeconomic status, smoking status, pre-pregnancy and current BMI, parity, family history of T2DM or cardiovascular disease, ethnicity, and time of delivery. Further research is needed to evaluate the value of embedding these pregnancy complications as part of assessment for future risk of T2DM and chronic hypertension.

**Supplementary Information:**

The online version contains supplementary material available at 10.1186/s12916-024-03284-4.

## Background

Pregnancy complications affect at least 30% of pregnancies and are among the leading causes of death in pregnancy [1, 2]. Physiological changes in the mother’s body during pregnancy that include increased cardiac output, a greater inflammatory response and metabolic abnormalities related to insulin resistance [3–6], can lead to some of these complications such as hypertensive disorders in pregnancy (HDP) and gestational diabetes mellitus (GDM). HDP affects between 3 and 10% of pregnancies and is the third cause of maternal mortality globally [1, 7, 8]. GDM affects around 7–10% of pregnancies globally and can lead to a higher incidence of still births and large for gestational-age babies [9].

Many of the pregnancy complications have an effect on the mother’s health and wellbeing beyond pregnancy. In a recent umbrella review, pregnancy-related complications were related to health conditions such as cardiovascular diseases [10], and results from systematic reviews have shown HDP and GDM were associated with risk of both T2DM and hypertension [11–17]. Although umbrella reviews that have synthesised the risk of hypertension and T2DM in women with a history of preeclampsia and GDM have been published [18–20], synthesis of the evidence for other pregnancy complications and a comprehensive review of the spectrum of HDP is lacking. Furthermore, there are new systematic reviews since the publication of these reviews and therefore these umbrella reviews need updating. The synthesis of this information would be beneficial to women, clinicians and other healthcare stakeholders to develop strategies to reduce the risk of future cardio-metabolic conditions in the postpartum period, a period which has been identified as a potential window of opportunity to prevent conditions associated with a history of pregnancy complications such as cardiovascular disease and diabetes [2, 21, 22].

This review aims to identify, appraise, synthesise, and consolidate evidence from systematic reviews and meta-analyses that have assessed the association between pregnancy-related complications and risk of post-partum hypertension and T2DM.

## Methods

The umbrella review was reported following Preferred Reporting Items for Overviews of Reviews (PRIOR) guidelines [23]. We also registered the protocol prior to the review in PROSPERO (registration No. CRD42022323718) and the protocol has undergone peer review [24].

### Exposures, comparator, and outcomes

Table 1 shows the pregnancy complications (exposures) we identified through discussion within the MuM-PreDiCT consortium [25] experts.

**Table 1:** List of pregnancy complications considered, key terms used to develop the search strategy and information extracted from systematic reviews

Pregnancy complications considered	Key terms used to develop the search strategy	Information extracted from systematic reviews
Pregnancy loss (miscarriage and stillbirth), hypertensive disorders of pregnancy (gestational hypertension, eclampsia, preeclampsia, HELLP (hemolysis, elevatated liver enzymes, and low platelets) syndrome), placental disorders (placenta previa, abruption, accreta, percreta), hyperemesis gravidarum, GDM, ectopic pregnancy, molar pregnancy, multiple pregnancy, obstetric haemorrhage, pre-term birth, mode of delivery, low birth weight (including small-for-gestational age, intra-uterine growth retardation, foetal growth restriction), post-partum depression, puerperal psychosis, perineal trauma, obstetric cholestasis and pelvic girdle pain.	Pregnancy complications OR pregnancy loss OR miscarriage OR stillbirth OR hypertensive disorders of pregnancy OR gestational hypertension OR eclampsia OR preeclampsia OR HELLP (hemolysis, elevated liver enzymes, and low platelets) syndrome OR placental previa OR placental abruption OR placenta accreta OR placenta percreta OR hyperemesis gravidarum OR gestational diabetes mellitus OR ectopic pregnancy OR molar pregnancy OR multiple pregnancy OR obstetric haemorrhage OR pre-term birth OR mode of delivery OR low birth weight OR small for gestational age OR intra-uterine growth retardation OR fetal growth restriction OR postpartum depression OR perineal psychosis OR pelvic girdle pain AND (type 2 diabetes OR hypertension).	Authors; year of publication; geographical area; aim of the review; databases searched; search period; population; health care setting; exposures; comparator; outcomes; covariates; study design; definitions of exposures and outcomes; data synthesis methods; quality assessment tool used; number of studies included in both qualitative and quantitative analysis; summary estimates and 95% confidence intervals and authors conclusions.

The comparator group was women who did not have the pregnancy complication of interest. The outcomes were T2DM and hypertension, diagnosed any time after pregnancy.

### Inclusion criteria

We included systematic reviews with or without meta-analysis investigating the association between any of the exposures defined previously and any of the outcomes defined previously in women with a history of pregnancy. We excluded narrative reviews, literature reviews, commentaries, conference abstracts, genetic studies and reviews looking at association between the exposures and T2DM and hypertension which were diagnosed during pregnancy.

### Search strategy

We searched Ovid Medline, Embase and Cochrane Database of Systematic Reviews from inception until 26 September 2022. There was no language restriction. We developed the search strategy around the key terms presented in Table 1.

We also limited the search to systematic reviews and meta-analyses using appropriate search filters and terms [26]. In addition, we searched the references of identified systematic reviews for further studies. The detailed search strategy for Ovid Medline database is provided in Additional file 1: Table S1. The search strategy was adapted for searches conducted in Embase and Cochrane databases.

### Study selection and data extraction

Two reviewers (SW and MS) independently reviewed all titles and abstracts identified by the search using Endnote and Covidence. Articles were included for full-text review if they met the inclusion criteria defined previously. In the case where there were any disagreements, these were resolved through discussion and where necessary with consultation with a third reviewer (FC). Data was extracted by the two reviewers independently using a data extraction form developed for this umbrella review. Table 1 presents information extracted from systematic reviews:

The form used for the data extraction was adapted from the Joanna Briggs Institute and is provided in Additional file 1: Table S2.

### Quality assessment

Methodological quality was assessed independently by two reviewers (SW and MS) using the AMSTAR 2 (A Measurement Tool to Assess Systematic Reviews) checklist [27]. In the case where there were any disagreements, these were resolved through discussion and where necessary through discussion with a third reviewer (FC). Seven of the sixteen items in the checklist are considered critical in determining the validity of a review [3]. The seven critical items are as follows: whether the review registered the protocol; whether the literature search was comprehensive; reasons for excluding individual studies were provided; risk of bias for individual studies was assessed (using recommended tools such as the Risk Of Bias In Non-randomized Studies of Interventions I (ROBINS-I) tool [28], Quality In Prognosis Studies (QUIPS) tool [29] or The Newcastle–Ottawa Scale (NOS) [30]); appropriate meta-analytical methods were used; whether the interpretation of results considered risk of bias in individual studies; and whether publication bias was assessed. We rated the reviews using gradings that are recommended by the AMSTAR 2 checklist authors: critically low, where more than one of the critical items was not satisfied with or without non-critical items being satisfied; low, where one critical item was not satisfied with or without non-critical items unsatisfied; moderate, where all critical items were satisfied and more than one non-critical items were not met; high, where all critical items were satisfied with no more than one non-critical item not met.

### Overlapping and outdated reviews

Two or more reviews that evaluated the same exposure(s) and outcome(s) were considered to be potentially overlapping if they included the same sets of primary studies [31, 32]. The degree of overlap was quantified by generating a citation matrix with systematic reviews as the columns and primary studies as the rows for each exposure-outcome combination of interest [32, 33]. A measure of overlap, the corrected covered area (CCA) was then calculated using the formula below,

$$CCA=\frac{(N-r)}{(rc-r)}$$

where *N* represents the number of publications included in evidence synthesis, *r* is the number of rows, and *c* is the number of columns. Overlap was considered very high if CCA was greater than 15%, high if between 11 and 15%, moderate if between 6 and 10% and slight if between 0 and 5% [32]. We used the following criteria to manage overlap between reviews [34–36]: (1) where the overlap occurred between Cochrane and non-Cochrane reviews, the Cochrane review was selected as previous studies have shown the evidence from Cochrane reviews is of higher quality [35]; (2) where CCA was more than 11%, and neither of the reviews was a Cochrane review, preference was given to the review that met the following criteria: (i) had highest AMSTAR 2 rating and the rating was moderate or higher; (ii) was most recent; had conducted a meta-analysis or provided pooled summary estimates; and (iii) had the greatest sample size (including both the number of studies and participants); (3) where CCA was less than 10%, all reviews were retained and the findings compared.

### Data synthesis

For associations that were reported in only one review, we used the effect size reported in the original meta-analysis (i.e. hazard ratio (HR), relative risk (RR), odds ratio (OR), or mean difference (MD)). For associations that were reported in more than one included review, we estimated ORs for each review using the reported data (i.e. number of controls, exposed and events in the included studies) to enable comparison across reviews [37]. Additionally, we performed a meta-analysis of primary studies included in the reviews after excluding duplicate studies across the reviews to obtain a summary estimate. We extracted information on the variables adjusted for in primary studies included in each systematic review. We extracted the definitions of exposures and outcomes provided in the corresponding systematic review or meta-analysis and reported them in a table. We also extracted data on the follow-up time of assessment of outcomes reported in each review and the confounders adjusted for in the primary studies included in each systematic review.

For each meta-analysis of the primary studies, summary effect size estimate and 95% confidence intervals (CI) were estimated using the inverse variance random-effects model using the DerSimonian and Laird method [38, 39]. Heterogeneity among included studies in each meta-analysis was assessed using the Cochrane *Q* test and reported using tau^2^ and inconsistency in the estimates was reported using I^2^ [40]. The 95% prediction interval (95% PI) for each meta-analysis was also estimated to evaluate the expected uncertainty in the effect estimates for a study evaluating the same association [41, 42]. Egger’s test was used to quantify small-study effects bias if more than 10 studies were included in the meta-analysis [43]. For studies where there were no outcomes in the exposed or control group, a continuity correction using 0.5 was applied to avoid any division by zero [44].

All statistical analyses were conducted using R [45], RStudio [46] and the meta package [47].

#### Deviations from protocol

There were no deviations from the protocol.

### Patient and public involvement

Patients and the public involvement representatives of the MuM-PreDiCT consortium [25] were involved in selecting the pregnancy complications. We plan to engage patient and public representatives, local policy makers in public health, and local charities (e.g. British Heart Foundation) to disseminate the results of the review in conferences and on social media.

## Results

### Literature search

The literature search yielded 6743 articles. After removing duplicates and screening titles and abstracts, 79 full-text articles were selected for further review. Fifty-nine articles were excluded after full-text screening, leaving 20 reviews included for evaluation. The list of excluded articles with reasons is provided in Additional file 1: Table S3 [13, 18–20, 48–110].

### Methodological quality

Using the AMSTAR 2 rating criteria, 8 of the 20 reviews were rated high quality [14, 15, 105, 111–115], 4 were moderate quality [16, 17, 116, 117], 5 were rated low quality [13, 102, 104, 106] and 3 were rated as critically low quality [99, 108, 110]. Assessment of the quality of the systematic reviews is presented in Additional file 1: Table S4. The three reviews of critically low quality were excluded from further evaluation, resulting in 17 reviews being taken forward for further evaluation.

### Overlapping and non-overlapping reviews

Ten of the 17 remaining reviews included similar sets of primary studies [13, 102, 104–107, 111]. These included the associations of two pregnancy complications (GDM and HDP) with risk of T2DM at any time after pregnancy. Among the overlapping reviews, the review selected was of moderate or high quality, was the latest and had the highest number of included primary studies, as described in the methods section.

Using the criteria for handling overlapping reviews, two of the 10 reviews with overlapping sets of primary studies were selected (one review for the association between GDM and T2DM and the second for the association between HDP and T2DM) [114, 115] and the other eight were considered for exclusion. The review selected for the association between GDM, and T2DM had the most primary studies among the overlapping reviews with at least moderate quality [114]. The second review for the association between HDP and T2DM was the latest, of high quality and had the most primary studies [115]. Additional file 1: Tables S5a to S5g show the citation matrices with the degree of overlap between reviews for the association between the two pregnancy complications and T2DM. Further, Additional file 1: Table S6 shows the general characteristics of reviews with overlapping primary studies and the calculated CCA with accompanying reasons for including or excluding them. Out of the eight reviews considered for exclusion, one review evaluated both T2DM and hypertension and was eligible for inclusion for the association between preeclampsia and hypertension and was therefore retained [117]. Therefore, in total seven reviews were excluded resulting in ten reviews remaining for further analysis. The list of the excluded reviews due to the overlap is provided in Additional file 1: Table S7 [102–107, 111].

### Study characteristics of reviews with non-overlapping studies

**Fig. 1:**
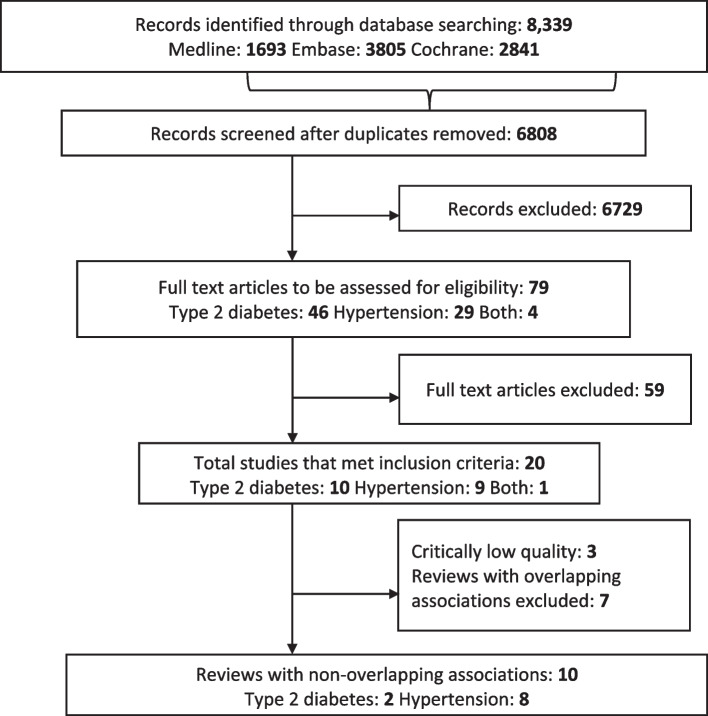
Flow diagram

In total, there were 10 systematic reviews included in this umbrella review. The flow diagram of these studies is shown in Fig. 1 below, and the general characteristics of the included reviews are provided in Table 2 below. The systematic reviews assessed two pregnancy complications. These were GDM and hypertensive disorders of pregnancy (including chronic hypertension, gestational hypertension, preeclampsia, eclampsia and HELLP syndrome). Additional file 1: Table S8 [14–17, 112–117] includes the definitions of the exposures and the extent of other confounders adjusted for in the reviews included. The number of women included in the systematic reviews ranged between 1,332,373 and 3,095,457 for studies relating to postpartum T2DM while for hypertension it was between 8041 and 2,711,443 (Table 2).

**Table 2:** General characteristics of systematic reviews included in the umbrella review

Author/year	Countries included in the reviews	Systematic review objective	Pregnancy complication	Comparator	Population source (number of participants in each meta-analysis/systematic review)	Outcome	Study designs (number of studies)	Quality appraisal tool	Funding source	AMSTAR 2 overall rating
Alonso-Ventura 2020 [16]	Australia, Brazil, Canada, Denmark, Finland, Iceland, Israel, Japan, Norway, Portugal, Sweden, the Netherlands, Turkey, UK, USA	To evaluate the association between preeclampsia and eclampsia on subsequent metabolic and biochemical outcomes.	Pre-eclampsia, eclampsia and HELLP syndrome	Women without Preeclampsia, eclampsia and HELLP syndrome. Women with uncomplicated pregnancies	Women with history of pregnancy (17,267)	Systolic blood pressure, diastolic blood pressure, hypertension	Cohort (41)	Newcastle–Ottawa Scale	None	Moderate
Bellamy 2007 [17]	Finland, Iceland, Jordan, New Zealand, Scotland, Spain, Sweden, UK, USA	To quantify the risk of future cardiovascular diseases, cancer, and mortality after preeclampsia.	Preeclampsia	Women who completed pregnancies without developing preeclampsia	Women with history of pregnancy (21,030)	Hypertension	Cohort (13)	The authors extracted relevant study characteristics for definition of exposure, outcome, sample size and degree of confounding, and used these in a sensitivity analysis	Department of Health’s National Institute for Health Research Biomedical Research Centre and British Heart Foundation	Moderate
Brown 2013 [116]	Brazil, Denmark, Finland, Iceland, Iran, Jordan, New Zealand, Norway, Portugal, Spain, Sweden, Thailand, UK, USA	To assess the current evidence and quantify the risks of cardiovascular disease (CVD), cerebrovascular events and hypertension associated with prior diagnosis of preeclampsia	Preeclampsia	Women who had uncomplicated/normotensive pregnancies	Women with history of pregnancy (82,255)	Hypertension	Cohort (30)	Authors used criteria based on how studies defined exposures and outcomes	Newcastle upon Tyne Hospitals NHS Foundation Trust.	Moderate
Dall'Asta 2021 [117]	Not provided	To elucidate whether preeclampsia and the gestational age at onset of the disease (early- vs. late-onset preeclampsia) have an impact on the risk of long-term maternal cardiovascular complications.	Preeclampsia	Women with previous normal pregnancy	Women with history of pregnancy (2,695,024)	Hypertension	Cohort (13) Case–control (2)	Newcastle–Ottawa Scale	Not provided	Moderate
Giorgione 2021 [14]	Canada, Israel, Kenya, Norway, Switzerland, The Netherlands, UK, USA	To estimate the incidence of hypertension in the first 2 years after Hypertensive disorders of pregnancy	Hypertensive disorders of pregnancy (gestational hypertension, preeclampsia, eclampsia, HELLP syndrome)	Women without hypertensive disorders of pregnancy during pregnancy	Women with history of pregnancy (8041)	Hypertension	Cohort (14) Case–control (1)	Newcastle–Ottawa Scale	European Union's 2020 research and innovation programme under the Marie Sklodowska-Curie grant	High
Pathirana 2021 [15]	Australia, Austria, Brazil, Canada, Chile, Denmark, Finland, Germany, Greece, Hong Kong, Hungary, India, Iran, Ireland, Italy, Korea, New Zealand, Norway, Poland, Senegal. South Korea, Spain, Sweden, Switzerland, Thailand, The Netherlands, UK, USA, Vienna	To synthesise evidence on conventional cardiovascular disease (CVD) risk factors among women with previous Gestational Diabetes Mellitus (GDM)	Gestational diabetes mellitus	Women with no history of gestational diabetes mellitus	Women with history of pregnancy (changes in systolic blood pressure as outcome = 50,118, changes in diastolic blood pressure as outcome = 49,495)	Systolic blood pressure, diastolic blood pressure	Cohort (27) Case–control (10) Cross-sectional (11)	Newcastle–Ottawa Scale	NHMRC Australia Public Health Early Career Fellowship and University of Adelaide	High
Sukmanee 2022 [113]	Australia, Canada, China, Denmark, Indonesia, Iran, Israel, Japan, Kenya, Korea, Netherlands, Norway, Singapore, South Africa, Spain, Sweden, Taiwan, Tanzania, UK, USA	To evaluate the risk of cardiovascular outcomes later in life in women with prior Hypertensive Disorders of Pregnancy in different years postpartum and in preeclamptic women with severe features, or early onset of preeclampsia.	Hypertensive disorders of pregnancy (gestational hypertension and preeclampsia, not chronic hypertension.)	Women with prior normotensive pregnancies, preeclampsia without severe features, or late-onset preeclampsia (developed after 34 completed weeks of gestation) depending on individual included studies	Women with history of pregnancy (215,528)	Hypertension	Cohort (37)	Newcastle–Ottawa Scale	Thailand Science Research and Innovation (TSRI) Research Career Development Grant and Prince of Songka University	High
Xu 2022 [112]	Brazil, Britain, China, France, Japan, Jordan, The Netherlands, Pakistan, Spain, Sweden, USA	To comprehensively evaluate the association between hypertensive disorders in pregnancy and the risk of developing chronic hypertension.	Hypertensive disorders of pregnancy or with the specific types of gestational hypertension and preeclampsia	Women without hypertensive disorders of pregnancy or specific types of preeclampsia and gestational hypertension	Women with history of pregnancy (hypertensive disorders of pregnancy as exposure = 228,317, preeclampsia as exposure = 341,060, gestational hypertension as exposure = 933)	Hypertension	Hypertensive disorders of pregnancy Cohort (5) Case–control (5) Cross-sectional (1) Gestational hypertension; cohort (1) Case–control (2) Preeclampsia; cohort (10) Case–control (3)	Newcastle–Ottawa Scale	National Natural Science Foundation of China	High
Vounzoulaki 2020 [114]	Australia, Canada, Finland, France, Hungary, India, Iran, Israel, Italy, Korea, Pakistan, Spain, Sri Lanka, Sweden, UK, USA	To estimate and compare progression rates to type 2 diabetes mellitus (T2DM) in women with gestational diabetes mellitus (GDM) and healthy controls	Gestational diabetes mellitus	Women with normoglycemic pregnancy	Women with history of pregnancy (1,332,373)	T2DM	Cohort (20)	Newcastle–Ottawa Scale	National Institute for Health Research	High
Zhao 2021 [115]	Australia, Canada, Denmark, Finland, Netherlands, Norway, Sweden, Taiwan, UK, USA	To quantify the association of previous Hypertensive Disorders of Pregnancy with incident diabetes mellitus	Hypertensive disorders of pregnancy (gestational hypertension, preeclampsia)	Women without hypertensive disorders of pregnancy	Women with history of pregnancy (number of participants not provided)	T2DM	Cohort; (hypertensive disorders of pregnancy (16), gestational hypertension (7), preeclampsia (11)	Newcastle–Ottawa Scale	None	High

### Summary findings

**Fig. 2:**
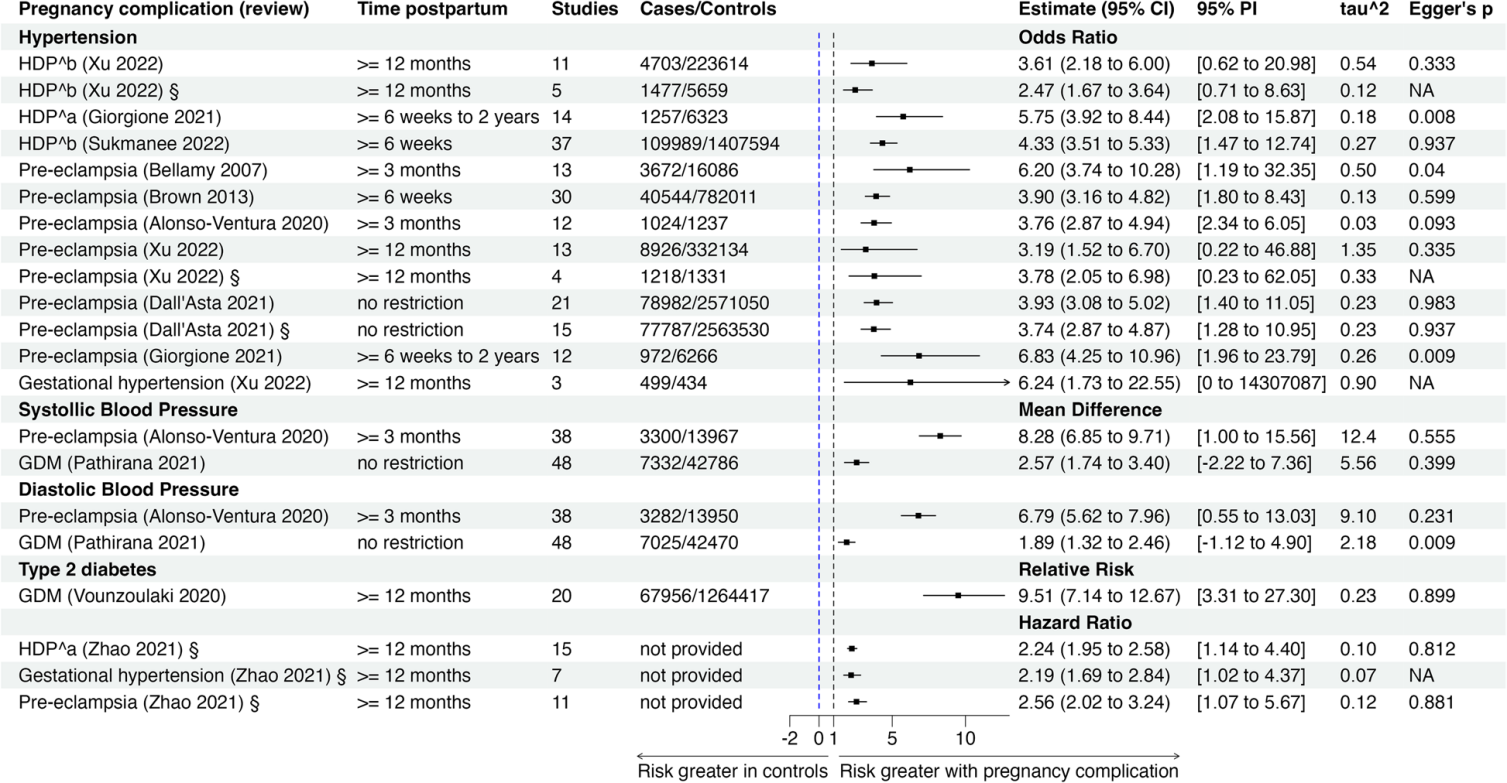
Forest plot of overall adjusted and unadjusted estimates of meta-analyses from systematic reviews that investigated the association between pregnancy complications and risk of future T2DM and hypertension. § Estimate from studies that adjusted for potential confounders. First vertical line is the reference point (zero) for the mean difference estimates. The second vertical line is the reference line (one) for the relative risk, odds ratio and hazard ratio estimates. Hazard ratios should be interpreted as an increase in the rate of the outcome while odds ratios should be interpreted as increase in the odds of the outcome and relative risk as an increase in the risk of the outcome in exposed compared to controls. For the association between hypertensive disorders of pregnancy (HDP) and hypertension, there were three reviews with non-overlapping associations and the estimates for each review are presented [14, 112, 113]. Similarly, five estimates from five reviews with non-overlapping associations between preeclampsia and hypertension are provided [16, 17, 112, 116, 117]. ^a^HDP included gestational hypertension and preeclampsia, including HELLP (haemolysis, elevated liver enzymes, lowered platelets) syndrome and eclampsia, and excluded pre-existing chronic hypertension. ^b^HDP included gestational hypertension and preeclampsia and excluded pre-existing chronic hypertension. Common adjustment factors across the studies were maternal age, body mass index, socioeconomic status, smoking status, pre-pregnancy and current BMI, parity, ethnicity and time of delivery Abbreviations: *GDM* gestational diabetes mellitus, *NA* not applicable, *95% PI* 95% prediction interval, *Egger’s p* Egger test *p* value

Overall and subgroup estimates alongside 95% CI for the associations of each pregnancy complication with T2DM and hypertension from the meta-analyses are shown in Table 3 below and the findings from narrative synthesis are shown in Additional file 1: Table S9 [15, 115]. A forest plot of the overall estimates is also provided in Fig. 2. Further estimates from subgroup analysis and publication bias measures are presented in Additional file 1: Table S10 [14–17, 112–117].

**Table 3:** Overall and subgroup/sensitivity analysis estimates from studies that carried out a meta-analysis on the association between pregnancy complications and risk of future T2DM and hypertension. Estimates (adjusted or unadjusted) presented alongside 95% confidence intervals, 95% prediction intervals, the number of studies included in the meta-analysis and the measures of inconsistency (*I*^2^) and heterogeneity (tau^2^)

Study	Exposure and adjustment factors in primary studies included in each systematic review	Outcome	Follow-up postpartum	Overall etimate (95% CI), prediction interval (PI), number of studies (studies), number of participants (*n*), inconsistency (*I*^2^), heterogeneity (tau^2^)	Subgroup and sensitivity analysis (estimate (95% CI), number of studies (studies), number of participants (*n*), inconsistency (*I*^2^), heterogeneity (tau^2^)
Alonso-Ventura 2020 [16]	Exposure: preeclampsia/eclampsia^c^ Common adjustment factors: **the review only provided information on matching variables for controls which were** gestational age, age, parity, time of delivery, ethnicity, weight, BMI, smoking habits and family history of DM, CVD and preeclampsia, contraceptive intake, alcohol consumption	Hypertension	Outcome measured at least 3 months after delivery	***Hypertension:* OR** 3.76 (2.87 to 4.94), PI = (2.34 to 6.05), studies = 12, *n* = 2261, *I*^2^ = 0%, tau^2^ = 0.03 ***SBP:*** MD 8.28 mmHg (6.85 to 9.71), PI = (1.00 to 15.56), studies = 38, *n* = 17,267, *I*^2^ = 78%, tau^2^ = 12.37 ***DBP:*** MD 6.79 mmHg (5.62 to 7.96), PI = (0.55 to 13.03), studies = 37, *n* = 17,232, *I*^2^ = 83%, tau^2^ = 9.10	**Hypertension** ***Time of follow-up*** ***Up to 5 years:*** OR 19.03 (2.47 to 146.49), studies = 2, *n* = 171, *I*^2^ = 0%, tau^2^ = 0 ***Up to 5 to 15 years:*** OR 4.13 (2.47 to 6.90), *n* = 1041**,** studies = 6, *I*^2^ = 0%, tau^2^ = 0.09 ***Above 15 years:*** OR 3.41 (2.41 TO 4.82), studies = 4, *n* = 1039, *I*^2^ = 0%, tau^2^ = 0.01 **SBP** ***Time of follow-up*** ***Up to 5 years:*** MD 9.88 (7.69 to 12.08), studies = 13, *n* = 1177, *I*^2^ = 58%, tau^2^ = 8.69 ***Up to 5 to 15 years:*** MD 6.82 (5.60 to 8.04), studies = 14, *n* = 3356, *I*^2^ = 0%, tau^2^ = 0 ***Above 15 years:*** MD 8.28 (4.99 to 11.58), studies = 11, *n* = 12,734, *I*^2^ = 92%, tau^2^ = 23.33 **DBP** ***Time of follow up*** ***Up to 5 years:*** MD 8.05 (6.50 to 9.60), studies = 12, *n* = 1142, *I*^2^ = 52%, tau^2^ = 3.53 ***Up to 5 to 15 years:*** MD 6.54 (4.85 to 8.23), studies = 14, *n* = 3356, *I*^2^ = 71%, tau^2^ = 6.30 ***Above 15 years:*** MD 5.78 (3.30 to 8.26), studies = 11, *n* = 12,734, *I*^2^ = 92%, tau^2^ = 14.24
Bellamy 2007 [17]	Exposure: preeclampsia^d^ Common adjustment factors: BMI, smoking, socioeconomic status, hypercholesterolaemia, type 2 diabetes	Hypertension	Outcome measured at least 3 months after delivery	OR 6.20 (3.74 to 10.28), PI = (1.19 to 32.35), studies = 13, *n* = 19,758, *I*^2^ = 83%%, tau^2^ = 0.50	***Parity*** ***Preeclampsia in any pregnancy:*** RR 5.96 (3.42 to 10.38), studies = 4, *n* = np, *I*^2^ = np, tau^2^ = np ***Preeclampsia in first pregnancy only:*** RR 3.23 (2.32 to 4.52), *n* = np, studies = 9, *I*^2^ = NP, tau^2^ = np
Brown 2013 [116]	Exposure: preeclampsia^d^ Common adjustment factors: Not provided	Hypertension	Outcome measured at least 6 weeks after delivery	OR 3.90 (3.16 to 4.82), PI = (1.80 to 8.43), studies = 30, *n* = 822,555, *I*^2^ = 80%, tau^2^ = 0.13	
Dall’Asta 2021 [117]	Exposure: preeclampsia^d^ Common adjustment factors: Not provided	Hypertension	No restriction	OR 3.93 (3.08 to 5.02), PI = (1.40 to 11.05), studies = 21, *n* = 2711443, *I*^2^ = 99%, tau^2^ = 0.23 OR_adj_ 3.74 (2.87 to 4.87), PI = 1.28 to 10.95, studies = 15, *n* = 2,695,024, *I*^2^ = 99%, tau^2^ = 0.23	
Giorgione 2021 [14]	Exposure: hypertensive disorders of pregnancy (including gestational hypertension, preeclampsia, eclampsia and HELLP syndrome)^a^ **Common adjustment factors:** not provided, however, the systematic review compares population statistics between controls and exposed groups by maternal age, ethnicity (black ethnicity), BMI and smoking	Hypertension	Outcome measured 6 weeks to 2 years after delivery	***HDP:*** OR 5.75 (3.92 to 8.44), PI = 2.08 to 15.87, studies = 14, *n* = 7580, *I*^2^ = 49%, tau^2^ = 0.18 **Preeclampsia:** OR 6.83 (4.25 to 10.96), PI = (1.96 to 23.79), studies = 12, *n* = 7238, *I*^2^ = 53%, tau^2^ = 0.26	**HDP: Follow-up duration** ***First 6 months:*** OR 13.39 (1.27 to 141.04), studies = 3, *n* = 392, *I*^2^ = 72%, tau^2^ = 3.05 ***6 months–1 year:*** OR 4.13 (2.82 to 6.07), studies = 3, *n* = 6741, *I*^2^ = 54%, tau^2^ = 0.06 ***1–2 years:*** OR 8.73 (4.66 to 16.35), studies = 8, *n* = 1364, *I*^2^ = 23%, tau^2^ = 0.18 **Preeclampsia: follow-up duration** ***First 6 months:*** OR 43.95 (5.72 to 338.04), studies = 2, *n* = 144, *I*^2^ = 0%, tau^2^ = 0 ***6 months–1 year:*** OR 4.46 (2.76 to 7.21), studies = 3, *n* = 5998, *I*^2^ = 56%, tau^2^ = 0.10 ***1–2 years:*** OR 8.91 (4.33 to 18.33), studies = 7, *n* = 1096, *I*^2^ = 33%, tau^2^ = 0.28 ***Estimates including cases with pre-existing chronic hypertension*** ***HDP:*** OR 6.28 (4.18 to 9.43), PI = (1.99 to 19.85), studies = 15, *n* = 8041, *I*^2^ = 56%, tau^2^ = 0.24 **Preeclampsia:** OR 7.49 (4.58 to 12.26), PI = (1.88 to 29.86), studies = 13, *n* = 7699, *I*^2^ = 60%, tau^2^ = 0.33 **HDP: follow up duration** ***First 6 months:*** OR 18.33 (1.35 to 249.48), studies = 3, *n* = 392, *I*^2^ = 84%, tau^2^ = 4.32 ***6 months–1 year:*** OR 4.36 (2.81 to 6.76), studies = 4, *n* = 7106, *I*^2^ = 56%, tau^2^ = 0.10 ***1–2 years:*** OR 7.24 (4.44 to 11.80), studies = 8, *n* = 1364, *I*^2^ = 9%, tau^2^ = 0.05 **Preeclampsia: follow up duration** ***First 6 months:*** OR 57.08 (11.00 to 296.07), studies = 2, *n* = 248, *I*^2^ = 0%, tau^2^ = 0 ***6 months–1 year***: OR 4.83 (2.78 to 8.37), studies = 4, *n* = 6307, *I*^2^ = 58%, tau^2^ = 0.16 ***1–2 years:*** OR 7.44 (4.19 to 13.21), studies = 7, *n* = 1144, *I*^2^ = 20%, tau^2^ = 0.12
Pathirana 2021 [15]	Exposure: gestational diabetes mellitus Common adjustment factors: BMI, parity, age, history of diabetes, other pregnancy complications	Hypertension	***No restriction***	**DBP:** MD 1.89 mmHg (1.32 to 2.46), PI = (− 1.12 to 4.90), studies = 48, *n* = 49,495, *I*^2^ = 83%, tau^2^ = 2.18 **SBP:** MD 2.47 mmHg (1.74 to 3.4), PI = (− 2.22 to 7.36), studies = 48, *n* = 50118, *I*^2^ = 79%, tau^2^ = 5.56	**Follow-up duration (DBP)** ** < *1 year:*** MD 2.48 mmHg (0.58 to 4.37), studies = np, *n* = 1749, *I*^2^ = 64%, tau^2^ = np ***1–5 years:*** MD 1.37 mmHg (0.20 to 2.54), studies = np, *n* = 19,676, *I*^2=^89%, tau^2^ = np ***5–10 years:*** MD 7.17 mmHg (1.69 to 16.03), studies = np,*n* = 2184, 99%, tau^2^ = np ** > *10 years:*** MD 1.23 mmHg (1.03 to 1.96), studies = np,*n* = 4948, *I*^2^ = 97%, tau^2^ = np **Follow-up duration (SBP)** ** < *1 year:*** MD 3.47 mmHg (1.26 to 5.68), studies = np, *n* = 1826, *I*^2^ = 50%, tau^2^ = np ***1–5 years:*** MD 2.26 mmHg (0.27 to 4.25), studies = np, *n* = 19,701, *I*^2^ = 93%, tau^2^ = np ***5–10 years:*** MD 3.96 mmHg (2.36 to 5.56), studies = np, *n* = 1965, *I*^2^ = 17%, tau^2^ = np ** > *10 years:*** MD 2.58 mmHg (1.05 to 4.11), studies = np, *n* = 4941, *I*^2^ = 23%, tau^2^ = np
Sukmanee 2022 [113]	Exposure: hypertensive disorders of pregnancy (including gestational hypertension and preeclampsia)^b^ Common adjustment factors: age, ethnicity, socioeconomic status, time of delivery, smoking status, BMI, family history of cardiovascular diseases, parity, history of diabetes mellitus and maternal education	Hypertension	Outcome measured at least 6 weeks after delivery	OR 4.33 (3.51 to 5.33), PI = (1.47 to 12.74), studies = 37, *n* = 1,517,583, *I*^2^ = 99%, tau^2^ = 0.27	***Follow-up duration*** ** ≤ *5 years:*** OR 6.05 (3.55 to 10.30), studies = 7, *n* = 321,971, *I*^2^ = 98%, tau^2^ = 0.35 ***6–10 years:*** OR 5.95 (4.69 to 7.54), studies = 6, *n* = 285,947, *I*^2^ = 82%, tau^2^ = 0.05 ***11–15 years:*** OR 4.22 (2.44 to 7.32), studies = 7, *n* = 786,479, *I*^2^ = 93%, tau^2^ = 0.41 ** > *15 years:*** OR 2.44 (1.93 to 3.08), studies = 2, *n* = 4535, *I*^2^ = 65%, tau^2^ = 0.02 ***Duration unspecified:*** OR 3.74 (2.50 to 5.58), studies = 15, *n* = 118,651, *I*^2^ = 97%, tau^2^ = 0.40 ***Preeclampsia with severe features*** OR 7.43 (2.85 to 19.35), studies = 4, *n* = 751,128, *I*^2^ = 90%, tau^2^ = 0.66
Xu 2022 [112]	Exposure: hypertensive disorders of pregnancy (including preeclampsia and gestational hypertension)^b^ Common adjustment factors: a list of adjustment factors in each primary study is not provided, however authors mention some studies adjusted for age and BMI at recruitment, pre-pregnancy BMI, age at first delivery and other factors	Hypertension	Included studies evaluating outcome at least 1 year after delivery	**HDP:** OR 3.61 (2.18 to 6.00), PI = (0.62 to 20.98), studies = 11, *n* = 228,317, *I*^2^ = 96%, tau^2^ = 0.84 HDP_adj_: OR 2.47 (1.67 to 3.64), PI = (0.71 to 8.63), studies = 5, *n* = 7137, *I*^2^ = 79%, tau^2^ = 0.12 ***Gestational hypertension:*** OR 6.24 (1.73 to 22.55), studies = 3, *n* = 933, *I*^2^ = 73%, tau^2^ = 0.90 ***Preeclampsia:*** OR 3.19 (1.52 to 6.70), PI = (0.22 to 46.88), studies = 13, *n* = 341,060, *I*^2^ = 97%, tau^2^ = 1.35 Preeclampsia_adj_: OR 3.78 (2.05 to 6.98), PI = (0.23 to 62.05), studies = 4, *n* = 2549, *I*^2^ = 90%, tau^2^ = 0.33	**HDP** **Region** **North and South America:** OR 2.11 (1.42 to 3.14), studies = 5, *I*^2^ = 82%, *n* = 6581, tau^2^ = 0.12 **Asia:** OR 4.26 (1.07 to 16.94), studies = 3, *n* = 1260, *I*^2^ = 88%, tau^2^ = 1.30 **Europe:** OR 5.52 (3.01 to 10.13), studies = 3, *n* = 220,476, *I*^2^ = 64%, tau^2^ = 0.17 **Test of differences between regions:** *p*-value = 0.03 **Preeclampsia** **Region** **North and South America:** OR 3.32 (1.26 to 8.74), studies = 6, *n* = 332,949, *I*^2^ = 95%, tau^2^ = 1.07 **Asia:** OR 7.54 (2.49 to 22.81), studies = 3, *n* = 6529, *I*^2^ = 36%, tau^2^ = 0.45 **Europe:** OR 2.19 (0.30 to 16.02), studies = 4, *n* = 1582, *I*^2^ = 98%, tau^2^ = 3.57
Vounzoulaki 2020 [114]	Exposure: gestational diabetes mellitus Common adjustment factors: maternal age, body mass index, family history of T2DM, parity, ethnicity, and socioeconomic status	T2DM	Included studies evaluating outcome at least 1 year after delivery	RR_unadj_ 9.51 (7.14 to 12.67), PI = (3.31 to 27.30), studies = 20, *n* = 1,332,373, *I*^2^ = 96.5%, tau^2^ = 0.23	***1. Ethnicity*** **White population:** RR 16.28 (15.01 to 17.66), studies = 6, *n* = 187,532, *I*^2^ = 0%, tau^2^ = 0 **Non-white population:** RR 10.38 (4.61 to 23.39), studies = 4, *n* = 2538, *I*^2^ = 78.2%, tau^2^ = 0.46 **Mixed populations:** RR 8.31 (5.44 to 12.69), studies = 10, *n* = 1,142,303, *I*^2^ = 97.8%, tau^2^ = 0.34 **Test of differences between ethnicities:** white vs mixed *p*-value = 0.26, white vs non-white *p*-value = 0.54) ***2. Length of follow-up*** **1 to 5 years:** RR 17.06 (8.95 to 32.55), studies = 6,* n* = 1566, *I*^2^ = 0%, tau^2^ = 0 ** > 5 to 10 years:** RR 10.42 (5.68 to 19.11), studies = 7, *n* = 223,945, *I*^2^ = 98%, tau^2^ = 0.52 ** > 10 years:** RR 8.09 (4.34 to 15.08), studies = 7, *n* = 1,106,862, *I*^2^ = 97%, tau^2^ = 0.52 **Test of differences between ethnicities:** 1–5 years vs 5–10 years *p*-value = 0.63, 1–5 years vs > 10 years *p*-value = 0.38)
Zhao 2021 [115]	Exposure: hypertensive disorders of pregnancy (HDP) (including gestational hypertension and preeclampsia)^a^ Common adjustment factors: maternal age, body mass index, socioeconomic status, smoking status, pre-pregnancy and current BMI, parity, ethnicity and time of delivery	T2DM	Included studies evaluating outcome at least 1 year after delivery	**HDP:** HR_adj_ 2.24 (1.95 to 2.58), PI = (1.14 to 4.40), studies = 15, *n* = 3,095,457, *I*^2^ = 94%, tau^2^ = 0.10 **Gestational hypertension:** HR_adj_ 2.19 (1.69 to 2.84), PI = (1.02 to 4.37) studies = 7, *n* = 2,735,586, *I*^2^ = 87%, tau^2^ = 0.07 **Preeclampsia:** HR_adj_: 2.56 (2.02 to 3.24), PI = (1.07 to 5.67), studies = 11, *n* = 3,007,543, *I*^2^ = 94%, tau^2^ = 0.23	**Preterm preeclampsia:** HR_adj_ 3.05 (2.05 to 4.56), studies = 3, *n* = np, *I*^2^ = 82%, tau^2^ = 0.09 **Follow-up duration** ***Less than 20 years:*** HR_adj_ 2.64 (2.23 to 3.12), studies = 7, *n* = np, *I*^2^ = 95%, tau^2^ = np ***Greater or equal to 20 years:*** HR_adj_ 1.34 (1.20 to 1.50), studies = 2, *n* = np, *I*^2^ = 0%, tau^2^ = np

adj — these are adjusted estimates otherwise they are unadjusted estimates

*Abbreviations*: *HR* hazard ratio, *RR* relative risk, *OR* odds ratio, *MD* mean difference, *GDM* gestational diabetes mellitus, *BMI* body mass index, *CVD* cardiovascular disease, *SBP* systolic blood pressure, *DBP* diastolic blood pressure, *NOS* Newcastle–Ottawa Scale, *np* data not provided

^a^Hypertensive disorders of pregnancy (HDP) included gestational hypertension and preeclampsia, including HELLP (haemolysis, elevated liver enzymes, lowered platelets) syndrome and eclampsia, and excluded pre-existing chronic hypertension

^b^HDP included gestational hypertension and preeclampsia, and excluded pre-existing chronic hypertension

^c^Preeclampsia excluded pre-existing chronic hypertension

^d^The systematic review did not mention whether pre-existing chronic hypertension was excluded from the analysis

### Pregnancy complications associated with T2DM

#### GDM

In a review of 20 primary studies including 1,332,373 women, the risk of T2DM at least 12 months after delivery was almost 10-fold greater in women with a history of GDM compared to healthy controls (RR 9.51, 95% CI 7.14 to 12.67, tau^2^ = 0.23) [114]. The summary estimate is based on unadjusted estimates (Table 3 and Fig. 2). Common confounders adjusted for by primary studies included in the review were maternal age, body mass index (BMI), family history of T2DM, parity, ethnicity, and socioeconomic status. All confounders adjusted for are presented in Additional file 1: Table S8, Tables S8a-S8f and Fig.S1.

#### Subgroup and sensitivity analysis

Analyses showed the association of GDM with risk of T2DM compared to those without GDM for women in subgroups defined by ethnicity remained elevated with the highest relative risk in women of white ethnicity and lowest in women of mixed ethnicity (white: RR 16.28, 95% CI 15.01 to 17.66, studies = 6, tau^2^ = 0); non-white: RR 10.38, 95% CI 4.61 to 23.39, studies = 4, tau^2^ = 0.46; mixed populations: RR 8.31, 95% CI 5.44 to 12.69, studies = 10, tau^2^ = 0.34). There were no statistically significant differences between the subgroups (white ethnicity vs mixed ethnicity, *p*-value = 0.26 and white ethnicity vs non-white ethnicity, *p*-value = 0.54). The association between GDM with risk of T2DM was attenuated over a longer period of follow-up (1 to 5 years: RR 17.06, 95% CI 8.95 to 32.55, studies = 6, tau^2^ = 0; more than 5 years and up to 10 years: RR 10.42, 95% CI 5.68 to 19.11, studies = 7, tau^2^ = 0.52; more than 10 years: RR 8.09, 95% CI 4.34 to 15.08, studies = 7, tau^2^ = 0.52). There were no statistically significant differences between the subgroups (1–5 years vs > 5–10 years, *p*-value = 0.63; 1–5 years vs > 10 years, *p*-value = 0.38). Subgroup estimates are provided in Table 3.

#### HDP

In the selected review, prior HDP (defined as gestational hypertension and preeclampsia, including HELLP (haemolysis, elevated liver enzymes, lowered platelets) syndrome and eclampsia) was associated with a higher rate of developing T2DM at least 1 year after delivery compared to those without those conditions (HR 2.24, 95% CI 1.95 to 2.58, studies = 15, tau^2^ = 0.10) (Table 3 and Fig. 2) [115]. The summary estimate is based on adjusted estimates.” The meta-analysis included a total of 3,095,457 women. Common factors adjusted for in the included primary studies include maternal age, BMI, socioeconomic status, smoking status, parity, ethnicity and time of delivery. All confounders adjusted for are presented in Additional file 1: Table S8, Table S8a-S8f and Fig.S1. A narrative review also reported that women with prior HDP developed T2DM at earlier ages than women without the pregnancy complication [115, 118] (Additional file 1: Table S9 [115]).

#### Subgroup and sensitivity analysis

Studies with shorter follow-up duration showed a higher rate of T2DM (less than 20 years of follow-up: HR 2.64, 95% CI 2.23 to 3.12, studies = 7; greater or equal to 20 years of follow-up: HR 1.34, 95% CI 1.20 to 1.50, studies = 2).

There was a dose–response relationship between HDP severity and future risk of T2DM (gestational hypertension: HR 2.19, 95% CI 1.69 to 2.84, studies = 7, tau^2^ = 0.07; preeclampsia: HR 2.56, 95% CI 2.02 to 3.24, studies = 11, tau^2^ = 0.23; preterm preeclampsia (preeclampsia that resulted in preterm birth < 37 weeks): HR 3.05, 95% CI 2.05 to 4.56, studies = 3, tau^2^ = 0.09). Subgroup estimates are provided in Table 3.

### Pregnancy complications associated with hypertension

#### GDM

The included review showed that women with previous GDM have higher mean systolic blood pressure (SBP) and diastolic blood pressure (DBP) any time after delivery compared to women without previous GDM (SBP: mean difference 2.47 mmHg, 95% CI 1.74 to 3.4, studies = 48, tau^2^ = 5.56); DBP: mean difference 1.89 mmHg, 95% CI 1.32 to 2.46, studies = 48, tau^2^ = 2.18). The summary estimates are based on unadjusted estimates (Table 3 and Fig. 2) [15]. BMI, parity, age, history of diabetes and other pregnancy complications were most adjusted for in included primary studies in the review. In the narrative synthesis of 12 primary studies, six reported higher mean DBP in women with previous GDM compared to the control group, with three studies showing statistical significance, and eight studies reported higher mean SBP with five studies showing statistical significance [15] Additional file 1: Table 9 [15].

#### Subgroup analysis

Further analysis by follow-up duration shows higher mean blood pressure in women with a history of GDM across different periods of follow-up, with the highest mean blood pressure observed for the period 5 to 10 years postpartum (*DBP:* < 1 year mean difference 2.48 mmHg, 95% CI 0.58 to 4.37; 1–5 years mean difference 1.37 mmHg, 95% CI 0.20 to 2.54; 5–10 years mean difference 7.17 mmHg, 95% CI 1.69 to 16.03; > 10 years mean difference 1.23 mmHg, 95% CI 1.03 to 1.96, *SBP:* < 1 year mean difference 3.47 mmHg, 95% CI 1.26 to 5.68; 1–5 years mean difference 2.26 mmHg, 95% CI 0.27 to 4.25; 5–10 years mean difference 3.96 mmHg, 95% CI 2.36 to 5.56; > 10 years mean difference 2.58 mmHg, 95% CI 1.05 to 4.11). Subgroup estimates are provided in Table 3.

#### HDP

Three reviews reported higher odds (3- to 6-fold higher) of hypertension in women with a history of HDP compared to women without a history of HDP [14, 112, 113]. Two of the three reviews defined HDP to include gestational hypertension and preeclampsia, excluding chronic hypertension [112, 113] while the third study defined HDP to include gestational hypertension, preeclampsia, eclampsia and HELLP syndrome (providing estimates with and without including chronic hypertension) [14].

In the two reviews, the odds of hypertension were significantly higher in the HDP group (OR 3.61, 95% CI 2.18 to 6.00, studies = 11, tau^2^ = 0.54, time = at least 1 year postpartum) and (OR 4.33, 95% CI 3.51 to 5.33, studies = 37, tau^2^ = 0.27, time = at least 6 weeks postpartum) and in the third review the reported risk remained higher between 6 weeks and 2 years postpartum both before excluding pre-existing chronic hypertension in the analysis (OR 6.28, 95% CI 4.18 to 9.43, studies = 15, tau^2^ = 0.24) and after excluding pre-existing chronic hypertension (OR 5.75, 95% CI 3.92 to 8.44, studies = 14, tau^2^ = 0.18) The summary estimate is based on unadjusted estimates (Table 3 and Fig. 2) [14]. Common confounders adjusted for in the primary studies included in the three reviews included age, ethnicity, socioeconomic status, BMI, family history of cardiovascular diseases, parity, history of diabetes mellitus and smoking status. The odds remained elevated after meta-analysing the primary studies that excluded chronic hypertension from the three systematic reviews (OR 4.26, 95% CI 3.54 to 5.12, studies = 53, tau^2^ = 0.29) (Additional file 1: Fig.S2).

On the specific types of HDP, six reviews reported between 3 and 10-fold higher in the odds of hypertension in women with a history of preeclampsia (OR 6.20, 95% CI 3.74 to 10.28, studies = 13, tau^2^ = 0.50; OR 3.90, 95% CI 3.16 to 4.82, studies = 30, tau^2^ = 0.13; OR 3.74, 95% CI 2.87 to 4.94, studies = 15, tau^2^ = 0.23; OR 3.19, 95% CI 1.52 to 6.70, studies = 13, tau^2^ = 1.35; OR 6.83, 95% CI 4.25 to 10.96, studies = 12, tau^2^ = 0.26; OR 3.76, 95% CI 2.87 to 4.94, studies = 12, tau^2^ = 0.03) (Table 3 and Fig. 2) [14, 16, 17, 112, 116, 117]. Mean blood pressure was higher in women with a history of preeclampsia compared to women without a history of the pregnancy complication (SBP: mean difference 8.28 mmHg, 95% CI 6.85 to 9.71, studies = 38, tau^2^ = 12.37; DBP: mean difference 6.79 mmHg, 95% CI 5.62 to 7.96, studies = 37, tau^2^ = 9.10) (Table 3 and Fig. 2) [16].

### Subgroup and sensitivity analysis

Two reviews showed the risk of hypertension after HDP remained elevated but attenuated by follow-up; (first 6 months: OR 13.39, 95% CI 1.27 to 141.04, studies = 3, tau^2^ = 3.05; 6 to 12 months: OR 4.13, 95% CI 2.82 to 6.07, studies = 3, tau^2^ = 0.06; 1 to 2 years: OR 8.73, 95% CI 4.66 to 16.35, studies = 8, tau^2^ = 0.18) [14], and (less or equal to 5 years: OR 6.05, 95% CI 3.55 to 10.30, studies = 7, tau^2^ = 0.35; 6 to 10 years: OR 5.95, 95% CI 4.69 to 7.54, studies = 6, tau^2^ = 0.05; 11 to 15 years: OR 4.22, 95% CI 2.44 to 7.32, studies = 7, tau^2^ = 0.41; greater than 15 years: OR 2.44, 95% CI 1.93 to 3.08, studies = 2, tau^2^ = 0.02; Duration unspecified: OR 3.74, 95% CI 2.50 to 5.58, studies = 15, tau^2^ = 0.40) [113].

Further subgroup analysis in one of the reviews showed the odds of post-partum hypertension in women with a history of HDP compared to healthy controls differed in different continents with the lowest odds in North and South America (OR 2.11, 95% CI 1.42 to 3.14, studies = 5, tau^2^ = 0.12), highest in Europe (OR 5.52, 95% CI 3.01 to 10.14, studies = 3, tau^2^ = 0.17) and similar to the overall estimate in Asia (OR 4.26, 95% CI 1.05 to 17.21, studies = 3, tau^2^ = 1.30) [112]. The differences among the continents were statistically significant (*p*-value = 0.03).

On the specific types of HDP, two reviews reported differences in odds of postpartum hypertension after preeclampsia by follow-up periods; first review: (first 6 months: OR 43.95, 95% CI 5.72 to 338.04, studies = 2, tau^2^ = 0; 6–12 months: OR 4.46, 95% CI 2.76 to 7.21, studies = 3, tau^2^ = 0.10; 1–2 years: OR 8.91, 95% CI 4.33 to 18.33, studies = 7, tau^2^ = 0.28) [14], second review: (up to 60 months: OR 19.03, 95% CI 2.47 to 146.49, studies = 2, tau^2^ = 0; up to 60.1 to 180 months: OR 4.13, 95% CI 2.47 to 6.90, studies = 6, tau^2^ = 0.09; above 180 months: OR 3.41, 95% CI 2.41 to 4.82, studies = 4, tau^2^ = 0.01) [16]. The odds of postpartum hypertension after preeclampsia was greater in women from Asia (OR 7.54, 95% CI 2.49 to 22.81, studies = 3, tau^2^ = 0.45) compared to women in North and South America (OR 3.32, 95% CI 1.26 to 8.74, studies = 6, tau^2^ = 1.07) and Europe (OR 2.19, 95% CI 0.30 to 16.02, studies = 4, tau^2^ = 3.57). There is however a lot of uncertainty in the estimates as the confidence intervals were quite wide and comparisons were made across studies rather than within. The subgroup estimates are provided in Table 3.

## Discussion

### Findings

In summary, results from this umbrella review showed the risk of T2DM at least 12 months after delivery was nearly 10-fold greater in women with previous GDM compared to women without GDM. Furthermore, mean blood pressure was higher in women with a history of GDM. HDP were associated with a higher rate of T2DM, and the risk of developing hypertension was 3- to 4-fold higher in women with a history of HDP or preeclampsia. It is noteworthy that the risk of hypertension was greater in women who had severe hypertensive disorders of pregnancy (e.g. severe preeclampsia and a preterm birth).

Analysis by follow-up based on one systematic review showed the risk of T2DM and hypertension after HDP and GDM was greatest in studies with shorter follow-up periods compared to those with longer periods of follow-up. The risk of T2DM in women with a history of GDM was highest in women of white ethnicity and lowest in women of mixed ethnicity, while the risk of hypertension in women with a history of HDP was highest in Europe and lowest in North and South America. However, for preeclampsia the risk of hypertension was highest in women from Asia compared to women in the Americas and Europe.

### Strengths and limitations

The umbrella review has the following strengths: registration of a protocol for the umbrella review prior to the start of the umbrella review; a comprehensive search of reviews in multiple databases; evaluation of the quality of the reviews using the recommended AMSTAR 2 checklist and including the quality of the reviews in the decision to include or exclude reviews in the evidence synthesis; assessment of overlap in reviews that included the same primary studies and reporting of relevant subgroup and sensitivity analyses.

The following limitations in the review could be considered in the interpretation of the results. Firstly, the number of participants, measures of heterogeneity and publication bias were not reported in some reviews. Secondly, some factors associated with the outcomes (e.g. ethnicity, lifestyle (diet, physical activity, smoking), family history of cardiovascular diseases etc.) were rarely included in the adjusted analyses. Thirdly, the strongest association between HDP and risk of hypertension was observed in the immediate post-partum period (< 6 months) in one systematic review. This may be an overestimate of risk and should be interpreted with caution because HDP may take up to 12 months to resolve [14, 119, 120]. Fourthly, although we obtained risk estimates for certain intervals of time in the postpartum period, it was difficult to obtain risk estimates at particular time points. Lastly, reviews on some pregnancy factors including miscarriage, pre-term birth and postnatal depression were not identified in the literature and hence not incorporated in this review.

### Methodological issues

There is potential for significant heterogeneity attributable to various study characteristics (e.g. study design and quality) as well as residual confounding inherent in observational studies. There is potential for recall bias for some of the results where primary studies included self-reported data and also misclassification of cases due to differences in diagnostic criteria by included primary studies, for instance, there is a possibility of misclassification of hypertension as pre-eclampsia or gestational hypertension, leading to overestimation of the effect sizes. Estimates from subgroup analyses with fewer than four studies should be interpreted with caution as the precision of the estimates might be affected where the number of studies included in a meta-analysis is small and heterogeneity is high [121, 122], and may be biased by confounding from other study-level characteristics. A more informative way to examine differences in subgroups is by conducting an individual participant meta-analysis [123].

### Comparison with other studies

Results from this review are consistent with previous research and guidelines showing that both HDP and GDM are associated with a higher risk of T2DM and hypertension. In 2018, a multiple exposure umbrella review for T2DM found one systematic review showing GDM was associated with almost 8-fold risk of T2DM [18], and in 2016, an umbrella review evaluating risk factors for vascular disease and mortality found two systematic reviews showing that both preeclampsia and gestational hypertension were associated with an elevated risk of hypertension [20]. A recent umbrella review which evaluated the relationship between preeclampsia and long-term maternal outcomes showed that preeclampsia was associated with a greater risk of developing both hypertension and diabetes mellitus [19].

### Potential mechanisms of association

Shared genetic factors between GDM and T2DM have also been hypothesised to be the link between the two conditions. Meta-analyses on the relationship between common T2DM genetic variants and GDM found nine variants shared between the two conditions on genes that may be related to β-cell function, insulin resistance and glycolysis [66, 124].

The mechanism through which HDP increases T2DM and hypertension is not well understood [115, 116] but may be mediated through insulin resistance [4, 125]. HDP may be an early manifestation of underlying insulin resistance as a result of increased metabolic demands during pregnancy, similar to GDM. Besides insulin resistance, other shared risk factors of HDP with cardiovascular diseases such as obesity, hypertension, hyperlipidaemia, and renal dysfunction may explain the link between HDP and T2DM [106, 126].

### Implications for practice, public health and future research

The findings of this review might help to increase awareness of the risk of these conditions to women and hence enable attendance at regular screening for hypertension and T2DM. Previous research has demonstrated risk factors for vascular conditions such as systolic blood pressure and total cholesterol reduced significantly for those who attended screening programmes for cardiovascular diseases [127].

Guidelines to prevent or mitigate the risk of future vascular conditions such as T2DM and hypertension recommend healthcare professionals to obtain detailed pregnancy history in women with a history of pregnancy complications to enable effective determination of the risk of future health conditions. The National Institute for Health and Care Excellence (NICE) guidelines recommend healthcare professionals to advise women with a history of HDP about their greater risk of hypertension and cardiovascular disease and encourage them to discuss with their GPs on ways to reduce the risk [128]. Avoiding smoking, maintaining a healthy lifestyle and a healthy weight are recommended in the guideline as potential interventions to reduce the risk of future hypertension. NICE guidelines also recommend risk assessment for T2DM in women with a history of GDM and no specific guidelines on risk assessment for women with a history of HDP [129].

Risk prediction models for diabetes (e.g. QDiabetes) and hypertension (e.g. Framingham score) in the general population have also been developed to calculate the risk of developing these conditions. However, QDiabetes does not include HDP and the Framingham score does not include both HDP and GDM in the risk equations [130, 131]. Further research is needed to quantify the prognostic value of adding a history of these pregnancy complications as predictors to these risk prediction models specifically in the postpartum period in the low-risk population of women of reproductive age who have a history of pregnancy.

## Conclusions

In summary, evidence from this umbrella review of systematic reviews showed strong and consistent evidence that women with previous GDM have a much greater risk of developing T2DM and higher blood pressure, and women with HDP had a greater risk of developing hypertension and T2DM. Further research is required to explain the underlying mechanisms of these associations and to evaluate whether adding these pregnancy complications to current risk prediction models improves the prediction of T2DM and hypertension in women with a history of pregnancy.

### Supplementary Information

**Additional file 1: Tables S1-11 and Figures S1-S2. Table S1 - **Search Strategy for systematic review,** Table S2** - Data extraction form, **Table S3** - List of excluded reviews, **Table S4** - AMSTAR 2 quality appraisal scores, **Table S5** - Citation matrices for reviews with overlapping associations, **Table S6** - General characteristics of reviews with overlapping associations, **Table S7** - List of reviews with overlapping associations excluded from analysis, **Table S8** - Exposure definitions and extend of risk factors adjustment in systematic reviews included in the umbrella review, **Table S9** - Tabular presentation of findings: Narrative syntheses, **Table S10** - Tabular presentation of original estimates: Meta-analysis, **Table S11** - PRIOR Checklist,** FigS1** - Word cloud of confounders adjustment reported by reviews, **FigS2** - meta-analysis of primary studies that excluded chronic hypertension.

## Data Availability

All data are included in this manuscript and its supplementary information files.

## Abbreviations

AMSTAR2: A MeaSurement Tool to Assess systematic Reviews 2
BMI: Body mass index
CCA: Corrected covered area
CI: Confidence intervals
CVD: Cardiovascular disease
DBP: Diastolic blood pressure
DM: Diabetes mellitus
GDM: Gestational diabetes mellitus
GP: General practitioner
HDP: Hypertensive disorders of pregnancy
HELLP: Hemolysis, elevatated liver enzymes, and low platelets
HR: Hazard ratio
MD: Mean difference
MuM-PreDiCT: Multimorbidity in Pregnancy: Determinants, clusters, Consequences and Trajectories
NHMRC: National Health and Medical Research Council
NHS: National Health Service
NICE: The National Institute for Health and Care Excellence
NOS: The Newcastle–Ottawa Scale
OR: Odds ratio
PRIOR: Preferred Reporting Items for Overviews of Reviews
QUIPS: Quality In Prognosis Studies
ROBINS-I: Risk Of Bias In Non-randomized Studies of Interventions I
RR: Relative risk
SBP: Systolic blood pressure
T2DM: Type 2 diabetes mellitus
