# Supplementary material for: Association between pregnancy-related complications and development of type 2 diabetes and hypertension in women: an umbrella review
Source: BMC Med. 2024 Feb 14;22:66. doi: 10.1186/s12916-024-03284-4 (PMC10865714; doi:10.1186/s12916-024-03284-4)
Supplement: Supplementary file 1 — Additional file 1: Tables S1-11 and Figures S1-S2. Table S1 - Search Strategy for systematic review, Table S2 - Data extraction form, Table S3 - List of excluded reviews, Table S4 - AMSTAR 2 quality appraisal scores, Table S5 - Citation matrices for reviews with overlapping associations, Table S6 - General characteristics of reviews with overlapping associations, Table S7 - List of reviews with overlapping associations excluded from analysis, Table S8 - Exposure definitions and extend of risk factors adjustment in systematic reviews included in the umbrella review, Table S9 - Tabular presentation of findings: Narrative syntheses, Table S10 - Tabular presentation of original estimates: Meta-analysis, Table S11 - PRIOR Checklist, FigS1 - Word cloud of confounders adjustment reported by reviews, FigS2 - meta-analysis of primary studies that excluded chronic hypertension. [file 12916_2024_3284_MOESM1_ESM.docx]

Table of Contents

[Supplementary materials 1](#_Toc157011386)

[Table S1: Search Strategy for systematic review 2](#_Toc157011387)

[Table S2: Data extraction form 3](#_Toc157011388)

[Table S3: List of excluded reviews 4](#_Toc157011389)

[Table S4: AMSTAR 2 quality appraisal scores 9](#_Toc157011390)

[Table S5: Citation matrices for reviews with overlapping associations 11](#_Toc157011391)

[Table S5a. Gestational Diabetes Mellitus and Type 2 Diabetes Mellitus 11](#_Toc157011392)

[Table S5b: Hypertensive Disorders of Pregnancy and Type 2 Diabetes Mellitus 13](#_Toc157011393)

[Table S5c: Pre-eclampsia and Type 2 Diabetes Mellitus 14](#_Toc157011394)

[Table S5d: Pre-eclampsia and Hypertension 15](#_Toc157011395)

[Table S5e: Hypertensive Disorders of Pregnancy and Hypertension 17](#_Toc157011396)

[Table S5f: Preeclampsia and Diastolic blood pressure 19](#_Toc157011397)

[Table S5g: Preeclampsia and systolic blood pressure 20](#_Toc157011398)

[Table S6: General characteristics of reviews with overlapping associations. 22](#_Toc157011399)

[Table S7: List of reviews with overlapping associations excluded from analysis 22](#_Toc157011400)

[Table S8: Exposure definitions and extend of risk factors adjustment in systematic reviews included in the umbrella review 24](#_Toc157011401)

[Table S8a: Extend of confounders adjustment in Alonso-Ventura 2020 review 30](#_Toc157011402)

[Table S8b: Extend of confounders adjustment in Bellamy 2007 review 32](#_Toc157011403)

[Table S8c: Extend of confounders adjustment in Pathirana 2021 review 33](#_Toc157011404)

[Table S8d: Extend of confounders adjustment in Sukmanee 2021 review 35](#_Toc157011405)

[Table S8e: Extend of confounders adjustment in Vounzoulaki 2021 review 39](#_Toc157011406)

[Table S8f: Extend of confounders adjustment in Zhao 2021 review 41](#_Toc157011407)

[FigS1: Word cloud of confounders adjustment reported by reviews. 45](#_Toc157011408)

[Table S9: Tabular presentation of findings: Narrative syntheses 46](#_Toc157011409)

[Table S10: Tabular presentation of original estimates: Meta-analysis 52](#_Toc157011410)

[FigS2: meta-analysis of the primary studies that excluded chronic hypertension from the three systematic reviews 59](#_Toc157011411)

[Table S11: PRIOR Checklist 60](#_Toc157011412)

# Supplementary materials

## Table S1: Search Strategy for systematic review

Ovid MEDLINE search

| **SNo.** | **Search term** | **Hits** |
| --- | --- | --- |
| 1 | pregnancy complication*.mp. or exp Pregnancy Complications/ | 467538 |
| 2 | exp Abortion, Spontaneous/ or ((recurrent adj3 miscarr$) or miscarr$ or early pregnancy loss$).mp. | 48594 |
| 3 | (stillbirth or still birth).mp. or exp Stillbirth/ or exp Fetal Death/ or (f?etal death$ or f?etal demise$).mp. | 41997 |
| 4 | Hypertension, Pregnancy-Induced/ or (gestational hypertension or (pregnancy adj3 hypertensi$)).mp. | 16348 |
| 5 | (preeclampsia or pre-eclampsia).mp. or exp Pre-Eclampsia/ | 49348 |
| 6 | exp Eclampsia/ or (eclampsia or tox?emia).mp. | 47057 |
| 7 | HELLP.mp. or exp HELLP Syndrome/ | 3231 |
| 8 | placenta accreta.mp. or exp Placenta Accreta/ or placenta percreta.mp. or placenta increta.mp. or morbidly adherent placenta.mp. or abnormally invasive placenta.mp. | 3884 |
| 9 | Placenta$ abruption.mp. or exp Abruptio Placentae/ | 4110 |
| 10 | placenta pr?eia.mp. or exp placenta previa/ or exp low lying placenta/ | 3253 |
| 11 | Hyperemesis Gravidarum.mp. or Hyperemesis Gravidarum/ or morning sickness.mp. or exp Morning Sickness/ | 2607 |
| 12 | (((pregnan$ or gestation$ or prenatal$ or antenatal$ or pre-natal$ or ante-natal$ or maternal$) adj2 diabet$) or gestational diabetes).mp. or exp Diabetes, Gestational/ or GDM.mp. | 35735 |
| 13 | ectopic pregnancy.mp. or exp Pregnancy, Ectopic/ or ((tub$$ adj3 pregnanc$) or (cornual adj3 pregnanc$) or (heterotopic adj3 pregnanc$) or (abdomin$ adj3 pregnanc$) or (extrauterine adj3 pregnanc$) or (interstitial adj3 pregnanc$) or (cervi$ adj3 pregnanc$) or (ovar$ adj3 pregnanc$) or (cesarean scar adj3 pregnanc$)).mp. | 25881 |
| 14 | exp Gestational Trophoblastic Disease/ or gestational trophoblastic.mp. or exp Hydatidiform Mole/ or ((hydatid? adj2 mole?) or (molar adj2 pregnanc?)).mp. | 7652 |
| 15 | exp Choriocarcinoma/ or choriocarcinoma.mp. | 9954 |
| 16 | exp Pregnancy, Multiple/ or ((pregnanc* or gestation*) adj (twin* or triplet* or quadruplet* or quintuplet* or multiple or multi?f?et*)).mp. or (Monochorionic or dichorionic).mp. | 15201 |
| 17 | exp Postpartum Hemorrhage/ or (postpartum hemorrhage or post partum hemorrhage or postpartum haemorrhage or post partum haemorrhage).ti,ab. or obstetric haemorrhage.mp. | 11867 |
| 18 | obstetric labor, premature.mp. or exp Obstetric Labor, Premature/ or (premature labor or premature labour or preterm labor or preterm labour or preterm birth).mp. | 45953 |
| 19 | exp Cesarean Section, Repeat/ or cesarean.mp. or exp Cesarean Section/ or (caesarean or cesarean or caesarian or cesarian or cesarien or caesarien or c-section or c section).mp. | 84405 |
| 20 | exp Extraction, Obstetrical/ or exp Obstetrical Forceps/ or ((operative or instrumental or assisted or forcep* or ventouse* or vacuum*) adj1 (deliver* or birth*)).mp. | 9032 |
| 21 | low birth weight.mp. or exp Infant, Low Birth Weight/ or (low birth weight* adj4 very low birth weight*).mp. | 52789 |
| 22 | exp Infant, Small for Gestational Age/ or small for gestational age.mp. or (small adj3 gestational age).mp. | 15417 |
| 23 | (intra?uterine growth adj2 (restriction* or retardation)).mp. or iugr.ti,ab. | 14160 |
| 24 | fetal growth retardation.mp. or exp Fetal Growth Retardation/ or (fetal growth adj2 (restriction? or retardation)).mp. | 21606 |
| 25 | postpartum depression.mp. or exp Depression, Postpartum/ | 8769 |
| 26 | ((postpartum* or post partum* or post-partum* or postnatal* or post natal* or post-natal* or perinatal* or peri natal* or peri-natal* or puerp*) and (depress* or dysthymi* or adjustment disorder* or mood disorder* or affective disorder*)).mp. | 19835 |
| 27 | (((postpartum* or post partum* or post-partum* or postnatal* or post natal* or post-natal or perinatal* or peri natal* or peri-natal* or puerp*) and (psychos#s or psychotic)) or psychosis after childbirth).mp. | 2038 |
| 28 | (((third or fourth or 3rd or 4th) adj degree) and tear*).mp. | 618 |
| 29 | (((anal near adj2 sphincter) or (rectal adj mucosa) or rectum or (anal adj epithelium) or anus or (recto?vaginal adj2 fistulae) or (anorectal adj mucosa) or (anal adj skin)) and (tear* or injur* or damage* or lacerat* or rupture* or trauma)).mp. [mp=title, book title, abstract, original title, name of substance word, subject heading word, floating sub-heading word, keyword heading word, organism supplementary concept word, protocol supplementary concept word, rare disease supplementary concept word, unique identifier, synonyms] | 6989 |
| 30 | ((obstetric* and anal and sphincter and injur*) or (anal and sphincter and injur*)).mp. | 2064 |
| 31 | (exp Pregnancy/ or exp Obstetrics/ or (pregnan* or obstetric*).mp.) and (exp Cholestasis/ or exp Cholestasis, Intrahepatic/) | 2086 |
| 32 | 1 or 2 or 3 or 4 or 5 or 6 or 7 or 8 or 9 or 10 or 11 or 12 or 13 or 14 or 15 or 16 or 17 or 18 or 19 or 20 or 21 or 22 or 23 or 24 or 25 or 26 or 27 or 28 or 29 or 30 or 31 | 621293 |
| 33 | exp Diabetes Mellitus, Type 2/ or diabetes type 2.mp. or Type 2/ or (MODY or NIDDM or T2DM or T2D).mp. or (non insulin* depend* or noninsulin* depend* or noninsulin?depend* or non insulin?depend*).mp. or ((typ? 2 or typ? II or typ?2 or typ?II) adj3 diabet*).mp. or (((late or adult* or matur* or slow or stabl*) adj3 onset) and diabet*).mp. | 234022 |
| 34 | exp hypertension/ or hypertens$.mp. or exp blood pressure/ or (blood pressure or bloodpressure).mp. | 880866 |
| 35 | 33 or 34 | 1076987 |
| 36 | systematic review.mp. | 273195 |
| 37 | (systematic$ adj2 (review$ or overview)).ti,ab. | 278575 |
| 38 | (systematic$ adj5 review$).tw,sh. | 294925 |
| 39 | meta-analysis.mp. or exp meta-analysis/ | 255317 |
| 40 | 36 or 37 or 38 or 39 | 426920 |
| 41 | 32 and 35 and 40 | 1693 |

## Table S2: Data extraction form

| **Field** |  |
| --- | --- |
| Study ID |  |
| Author/s |  |
| Year of publication |  |
| Geographical area |  |
| Aim of the review |  |
| Database searched |  |
| Search period |  |
| Population |  |
| Heath care setting |  |
| Exposures |  |
| Comparator |  |
| Outcomes |  |
| Covariates |  |
| Study design(s) |  |
| Definition of exposure |  |
| Definition of outcome |  |
| Data synthesis method |  |
| Quality assessment tool |  |
| Quality of the included primary studies as assessed by review authors |  |
| Number of studies included in qualitative analysis (narrative synthesis where meta-analysis was not done / possible by the review authors) |  |
| Number of meta-analyses |  |
| Number of studies included in each meta-analysis |  |
| Summary estimates of each meta-analysis and its related 95% confidence intervals |  |
| Author’s conclusion |  |
| Review limitation |  |
| Additional comments |  |

## Table S3: List of excluded reviews

| **1st Author and Year** | **Title** | **Reason for exclusion** |
| --- | --- | --- |
| Bellou 2018 | Risk factors for type 2 diabetes mellitus: An exposure-wide umbrella review of meta-analyses | Not a systematic review (Umbrella review) |
| Bundhun 2017 | Impact of systemic lupus erythematosus on maternal and fetal outcomes following pregnancy: A meta-analysis of studies published between years 2001-2016 | Exposure not in our list |
| DiCianni 2010 | Gestational diabetes mellitus: an opportunity to prevent type 2 diabetes and cardiovascular disease in young women | Not a systematic review |
| EdstedtBonamy 2013 | Predicting Women's Future Cardiovascular Health from Pregnancy Complications | Outcomes not T2DM or Hypertension |
| Gortner 2007 | Intrauterine growth restriction and risk for arterial hypertension: a causal relationship? | Not a systematic review |
| Guo 2016 | Postpartum Lifestyle Interventions to Prevent Type 2 Diabetes Among Women with History of Gestational Diabetes: A Systematic Review of Randomized Clinical Trials | Intervention between exposure and outcome |
| Hutchesson 2022 | Do modifiable risk factors for cardiovascular disease post-pregnancy influence the association between hypertensive disorders of pregnancy and cardiovascular health outcomes? A systematic review of observational studies | Outcomes not T2DM or Hypertension |
| Jenabi 2021 | The association between preeclampsia and the risk of metabolic syndrome after delivery: a meta-analysis | Outcomes not T2DM or Hypertension |
| kramer 2019 | Gestational diabetes and the risk of cardiovascular disease in women: a systematic review and meta-analysis | Outcomes not T2DM or Hypertension |
| Lao 2015 | The mother - The long-term implications on metabolic and cardiovascular complications | Not a systematic review |
| Lassi 2013 | Risk factors and interventions related to maternal and pre-pregnancy obesity, pre-diabetes and diabetes for maternal, fetal and neonatal outcomes: A systematic review | Outcomes during pregnancy |
| Leya 2013 | Association of plasma glucose and type 2 diabetes susceptibility genes with gestational diabetes | Not a systematic review |
| Li 2018 | Sex hormone-binding globulin, cardiometabolic biomarkers, and gestational diabetes-a longitudinal pregnancy cohort study and a meta-analysis | Exposure not in our list |
| Li 2016 | Parity and risk of type 2 diabetes: A systematic review and dose-response meta-analysis | Parity not in the list of exposures |
| Li 2014 | Preterm birth is associated with risk of essential hypertension in later life | Population not pregnant women |
| Li 2019 | Parity and risk of maternal cardiovascular disease: A dose-response meta-analysis of cohort studies | Exposure not in our list |
| Liao 2020 | Association of Low Birth Weight and Premature Birth With the Risk of Metabolic Syndrome: A Meta-Analysis | Population not pregnant women |
| Lin 2018 | Association of type 2 diabetes susceptible genes GCKR, SLC30A8, and FTO polymorphisms with gestational diabetes mellitus risk: a meta-analysis | Outcomes not T2DM or Hypertension |
| Liu 2020 | Lack of association between IGF2BP2 rs4402960 polymorphism and gestational diabetes mellitus: a case-control study, meta-analysis and trial sequential analysis | Outcomes not T2DM or Hypertension |
| Mao 2012 | Meta-analysis of the relationship between common type 2 diabetes risk gene variants with gestational diabetes mellitus | Outcomes not T2DM or Hypertension |
| McIntyre 2020 | Gestational Diabetes Mellitus-Innovative Approach to Prediction, Diagnosis, Management, and Prevention of Future NCD-Mother and Offspring | Not a systematic review |
| Minhas 2020 | The Association of Adverse Pregnancy Outcomes and Cardiovascular Disease: Current Knowledge and Future Directions | Not a systematic review |
| Moore 2021 | Association between the antepartum oral glucose tolerance test and the risk of future diabetes mellitus among women with gestational diabetes: A systematic review and meta-analysis | No controls |
| Morton 2014 | Interventions to modify the progression to type 2 diabetes mellitus in women with gestational diabetes: a systematic review of literature | Intervention between exposure and outcome |
| Neiger 2017 | Long-term effects of pregnancy complications on maternal health: A review | Not a systematic review (no search strategy) |
| Noctor 2019 | Risk associated with development of type 2 DM in gestational diabetes | Talk |
| Park 2020 | Contemporary clinical updates on the prevention of future cardiovascular disease in women who experience adverse pregnancy outcomes | Not a systematic review |
| Peacock 2014 | A review of interventions to prevent Type 2 Diabetes after Gestational Diabetes | Not a systematic review |
| Pedersen 2017 | How to prevent type 2 diabetes in women with previous gestational diabetes? A systematic review of behavioural interventions | Intervention between exposure and outcome |
| Pittara 2021 | Pre-eclampsia and long-term health outcomes for mother and infant: an umbrella review | Not a systematic review (Umbrella review) |
| Pons 2010 | A systematic review with meta-analysis of women with gestational diabetes indicate that they are at risk to suffer diabetes seven times more than normoglycemic women: Commentary | Commentary |
| Reddy 2019 | Preeclampsia and long-term cardiovascular function - A systematic review and meta-analysis | Outcomes not T2DM or Hypertension |
| Sangtani 2019 | Effect of Hypertensive Disorders of Pregnancy on Clinical Outcomes in Peripartum Cardiomyopathy | Population not pregnant women |
| Steinthorsdottir 2020 | Genetic predisposition to hypertension is associated with preeclampsia in European and Central Asian women | Not a systematic review |
| Theilen 2018 | Pre-eclampsia and cardiovascular risk: comparing apples with apples | Commentary |
| Tranidou 2020 | Prevalence of metabolic syndrome after pregnancies complicated by gestational diabetes mellitus - a systematic review and meta-analysis | Abstract |
| Tranidou 2021 | Risk of developing metabolic syndrome after gestational diabetes mellitus - a systematic review and meta-analysis | Outcomes not T2DM or Hypertension |
| Varner 2017 | Pregnancies After the Diagnosis of Mild Gestational Diabetes Mellitus and Risk of Cardiometabolic Disorders | Not a systematic review |
| Visser 2014 | Systematic review and metaanalysis on nonclassic cardiovascular biomarkers after hypertensive pregnancy disorders | Outcomes not T2DM or Hypertension |
| Wang 2013 | Incidence of adverse outcomes associated with gestational diabetes mellitus in low- and middle-income countries | Outcomes not T2DM or Hypertension |
| WenLo 2020 | Future cardiovascular disease risk for women with gestational hypertension: A systematic review and meta-analysis | Outcomes not T2DM or Hypertension |
| Xu 2014 | Metabolic syndrome risk after gestational diabetes: A systematic review and meta-analysis | Outcomes not T2DM or Hypertension |
| Zhao 2020 | 47 - Risk of Type 2 Diabetes Mellitus After Hypertensive Disorders of Pregnancy: A Systematic Review and Meta-Analysis | Abstract |
| Baptiste-Roberts 2009 | Risk Factors for Type 2 Diabetes Among Women with Gestational Diabetes: A Systematic Review | No controls |
| Ben-Haroush 2004 | Epidemiology of gestational diabetes mellitus and its association with Type 2 diabetes | Narrative synthesis (No quality assessement) |
| Burger 2022 | Hypertensive Disorders of Pregnancy and Cardiovascular Disease Risk Across Races and Ethnicities: A Review | No quality assessement |
| Cao 2022 | Adverse pregnancy outcomes are associated with an increased risk of postpartum prediabetes and diabetes in Chinese women with gestational diabetes | No controls |
| Craici 2008 | Pre-eclamptic pregnancies: An opportunity to identify women at risk for future cardiovascular disease | Priority paper evaluation |
| Farahvar 2019 | Gestational diabetes risk factors and long-term consequences for both mother and offspring: a literature review | Expert commentary |
| Groenhof 2017 | Preventing cardiovascular disease after hypertensive disorders of pregnancy: Searching for the how and when | Controls from a survey. No MA |
| Kim 2002 | Gestational diabetes and the incidence of type 2 diabetes: a systematic review | No controls |
| Krome 2016 | Gestational diabetes: Individual risk assessment for type 2 diabetes | Commentary |
| Leslie 2016 | Preeclampsia and the Risk of Future Vascular Disease and Mortality: A Review | Not a systematic review (Umbrella review) |
| Rayanagoudar 2016 | Quantification of the type 2 diabetes risk in women with gestational diabetes: a systematic review and meta-analysis of 95,750 women | No controls |
| Switzer 2022 | Hypertensive Disordersin Pregnancy and Future Risk of Stroke and Hypertension: A Systematic Review and Meta-analysis | Conference abstract |
| Veiga 2021 | Previous preeclampsia and its association with the future development of cardiovascular diseases: a systematic review and meta-analysis | Duplicate of study ID 13 |
| Wu 2017 | Preeclampsia and Future Cardiovascular Health: A Systematic Review and Meta-Analysis | Outcomes not T2DM or Hypertension |
| Bellamy 2009 | Type 2 diabetes mellitus after gestational diabetes: a systematic review and meta-analysis. | Due to overlap |
| Brouwers 2018 | Recurrence of pre-eclampsia and the risk of future hypertension and cardiovascular disease: a systematic review and meta-analysis | Controls not in PICO |
| Dennison 2021 | The absolute and relative risk of type 2 diabetes after gestational diabetes: A systematic review and meta-analysis of 129 studies | Due to overlap |
| Gadve 2021 | Risk of Developing Type 2 Diabetes Mellitus in South Asian Women with History of Gestational Diabetes Mellitus: A Systematic Review and Meta-Analysis. | Due to overlap |
| Li 2020 | Incidence Rate of Type 2 Diabetes Mellitus after Gestational Diabetes Mellitus: A Systematic Review and Meta-Analysis of 170,139 Women | No controls |
| Song 2018 | Long-term risk of diabetes in women at varying durations after gestational diabetes: a systematic review and meta-analysis with more than 2 million women | Due to overlap |
| Wang 2017 | Hypertensive disorders during pregnancy and risk of type 2 diabetes in later life: a systematic review and meta-analysis | Due to overlap |
| Wu 2016 | Pre-eclampsia is associated with a twofold increase in diabetes: a systematic review and meta-analysis | Due to overlap |
| You 2021 | Risk of type 2 diabetes mellitus after gestational diabetes mellitus: A systematic review & meta-analysis | Due to overlap |
| Hopmans 2015 | Increased risk of type 2 diabetes mellitus and cardiovascular disease after diabetes gravidarum. | Critically low quality |
| Cavalli 2021 | P-099. Previous preeclampsia and its association with future cardiovascular diseases: A systematic review and meta-analysis. | Critically low quality |
| Wu 2020 | Hypertensive Disorders of Pregnancy and Risk of Cardiovascular Disease-Related Morbidity and Mortality: A Systematic Review and Meta-Analysis. | Critically low quality |

## Table S4: AMSTAR 2 quality appraisal scores

| **Authors** | **1** | **2** | **3** | **4** | **5** | **6** | **7** | **8** | **9** | **10** | **11** | **12** | **13** | **14** | **15** | **16** | **Rating** |
| --- | --- | --- | --- | --- | --- | --- | --- | --- | --- | --- | --- | --- | --- | --- | --- | --- | --- |
| Alonso-Ventura 2020 | Yes | No | Yes | Yes | Yes | Yes | Partial Yes | Yes | Yes | No | Yes | Yes | Yes | Yes | Yes | Yes | Moderate |
| Bellamy 2009 | Yes | Partial Yes | Yes | Yes | Yes | Yes | Yes | Yes | No | No | Yes | Yes | Yes | Yes | Yes | Yes | Low |
| Bellamy 2007 | Yes | Partial Yes | Yes | Yes | Yes | Yes | Partial Yes | Yes | Partial Yes | No | Yes | Yes | Yes | Yes | Yes | Yes | Moderate |
| Brown 2013 | Yes | Partial Yes | Yes | Yes | No | Yes | Yes | Yes | Partial Yes | No | Yes | Yes | Yes | Yes | Yes | Yes | Moderate |
| Cavalli 2021 | Yes | Yes | No | Yes | Yes | Yes | Partial Yes | Yes | No | No | Yes | No | No | Yes | No | Yes | Critically Low |
| Dall'Asta 2021 | Yes | Yes | Yes | Yes | Yes | Yes | Yes | Yes | Yes | No | Yes | Yes | Yes | Yes | Yes | No | Moderate |
| Dennison 2021 | Yes | Yes | Yes | Yes | Yes | Yes | Partial Yes | Yes | Yes | No | Yes | Yes | No | Yes | No | Yes | Low |
| Gadve 2021 | Yes | Yes | Yes | Yes | Yes | Yes | Partial Yes | Yes | Yes | No | Yes | No | Yes | Yes | Yes | Yes | High |
| Giorgione 2021 | Yes | Yes | Yes | Yes | Yes | Yes | Partial Yes | Yes | Yes | No | Yes | Yes | Yes | Yes | Yes | Yes | High |
| Hopmans 2015 | Yes | Partial Yes | Yes | Yes | No | No | Partial Yes | No | Yes | No | Yes | Yes | Yes | No | No | Yes | Critically Low |
| Xu 2022 | Yes | Partial Yes | Yes | Partial Yes | Yes | Yes | Partial Yes | Yes | Yes | No | Yes | Yes | Yes | Yes | Yes | Yes | High |
| Sukmanee 2022 | Yes | Yes | Yes | Yes | Yes | Yes | Partial Yes | Yes | Yes | No | Yes | Yes | Yes | Yes | Yes | Yes | High |
| Pathirana 2021 | Yes | Yes | Yes | Yes | Yes | Yes | Partial Yes | Yes | Yes | No | Yes | Yes | Yes | Yes | Yes | Yes | High |
| Song 2018 | Yes | No | Yes | Partial Yes | Yes | Yes | Partial Yes | Yes | Yes | No | Yes | Yes | Yes | Yes | Yes | Yes | Low |
| Vounzoulaki 2020 | Yes | Yes | Yes | Yes | Yes | Yes | Partial Yes | Yes | Yes | No | Yes | Yes | Yes | Yes | Yes | Yes | High |
| Wang 2017 | Yes | Yes | Yes | Yes | Yes | Yes | Partial Yes | Yes | Yes | No | Yes | No | No | Yes | Yes | Yes | High |
| Wu 2016 | Yes | No | Yes | Yes | Yes | Yes | Partial Yes | Yes | Yes | No | Yes | Yes | Yes | No | Yes | Yes | Low |
| Wu 2020 | Yes | No | Yes | Partial Yes | Yes | Yes | Partial Yes | Yes | Yes | No | Yes | Yes | No | Yes | Yes | Yes | Critically Low |
| You 2021 | Yes | No | Yes | Partial Yes | Yes | Yes | Partial Yes | Yes | Yes | No | Yes | Yes | Yes | Yes | Yes | Yes | Low |
| Zhao 2021 | Yes | Yes | Yes | Yes | Yes | Yes | Partial Yes | Yes | Yes | No | Yes | Yes | Yes | Yes | Yes | Yes | High |

No MA= No meta-analysis. Item 1: inclusion of PICO elements? Item 2: review methods established before conduct of review? Item 3: explanation for selection of study designs to be included in review? Item 4: use of a comprehensive search strategy? Item 5: selection of studies in duplicate? Item 6: data extraction in duplicate? Item 7: provision of list of excluded studies with justification for exclusion? Item 8: description of included studies in adequate detail? Item 9: satisfactory technique for risk of bias? Item 10: sources of funding for included studies reported? Item 11: proper methods for metaanalysis? Item 12: potential risk of bias in included studies discussed? Item 13: risk of bias accounted for in interpreting results? Item 14: heterogeneity discussed? Item 15: if meta-analysis conducted was publication bias discussed? Item 16: disclosure of funding or conflict of interest?

## Table S5: Citation matrices for reviews with overlapping associations

### Table S5a. Gestational Diabetes Mellitus and Type 2 Diabetes Mellitus

| **Systematic review** | **Bellamy 2009** | **Dennison 2021** | **Gadve 2021** | **Song 2018** | **Vounzoulaki 2020** | **You 2021** |
| --- | --- | --- | --- | --- | --- | --- |
| **Overlapping associations** | **T2DM** | **T2DM** | **T2DM** | **T2DM** | **T2DM** | **T2DM** |
| **Primary study** |  |  |  |  |  |  |
| Aberg 2002 | X |  |  | X | X | X |
| Ajala 2015 |  |  |  |  |  | X |
| Akinci 2010 |  | X |  | X |  |  |
| Albareda 2003 | X | X |  | X | X | X |
| Anderberg 2011 |  |  |  | X |  |  |
| Anderberg 2012 |  | X |  |  |  | X |
| Aroda 2015 |  | X |  |  |  |  |
| Aziz 2018 |  |  | X |  | X |  |
| Barden 2013 |  | X |  |  |  | X |
| Benjamin 1993 | X |  |  |  |  | X |
| Bian 2000 | X |  |  | X |  | X |
| Bo 2004 |  |  |  | X |  |  |
| Bo 2006 |  | X |  |  |  |  |
| Bond 2017 |  | X |  |  |  |  |
| Carr 2006 |  | X |  | X |  |  |
| Chodick 2010 |  | X |  | X | X |  |
| Cormier 2015 |  |  |  |  |  | X |
| Corrado 2007 |  | X |  |  |  |  |
| Daly 2018 |  | X |  |  | X | X |
| Damm 1994 | X |  |  | X |  | X |
| Dornhorst 1990 |  | X |  |  |  |  |
| Engeland 2011 |  | X |  |  |  |  |
| Feig 2008 | X |  |  | X |  | X |
| Feig 2013 |  |  |  |  |  | X |
| Fengyun 2010 |  |  |  | X |  |  |
| Ferraz 2007 | X |  |  | X |  |  |
| Gabaldi Silva 2003 |  | X |  |  |  |  |
| Gadgil 2017 |  |  |  | X |  |  |
| Gar 2018 |  | X |  |  |  |  |
| Gobl 2011 |  | X |  |  |  |  |
| Goueslard 2016 |  | X |  | X |  |  |
| Gunderson 2007 | X |  |  | X |  |  |
| Hakkarainen 2015 |  |  |  |  |  | X |
| Han 2018 |  | X |  |  |  |  |
| Hanson 1996 |  | X |  |  |  |  |
| Herath 2017 |  | X | X |  | X | X |
| Hummel 2013 |  | X |  |  |  | X |
| Huopio 2004 |  | X |  |  | X |  |
| Ijas 2013 |  | X |  |  |  |  |
| Jarvela 2006 | X | X |  | X |  | X |
| Kaul 2015 |  | X |  | X |  |  |
| Ko 1999 | X |  |  | X |  | X |
| Kramer 2014 |  | X |  |  |  |  |
| Krishnaveni 2007 | X |  | X | X | X |  |
| Lauenborg 2005 |  | X |  | X |  |  |
| Lee 1994 |  | X |  |  |  |  |
| Lee 2007 | X | X |  | X | X |  |
| Lee 2008 | X |  |  |  | X | X |
| Linne 2002 | X |  |  | X | X | X |
| Lowe 2018 |  | X |  |  |  |  |
| Madarasz 2008 | X |  |  |  |  |  |
| Madarasz 2009 |  |  |  | X | X |  |
| Mai 2014 |  |  |  |  |  | X |
| Mai 2015 |  | X |  |  |  |  |
| Minooee 2017 |  | X |  |  | X |  |
| Moleda 2016 |  | X |  |  |  |  |
| Morimitsu 2007 | X |  |  | X |  | X |
| Mukerji 2012 |  |  | X |  | X | X |
| Nocter 2016 |  | X |  |  |  |  |
| O'Sullivan 1991 | X |  |  |  |  | X |
| Osei 1998 | X |  |  |  |  | X |
| Persson 1991 | X |  |  | X |  | X |
| Persson 2015 |  | X |  | X |  |  |
| Pintaudi 2015 |  | X |  |  | X | X |
| Pirkola 2009 |  |  |  | X |  |  |
| Pirkola 2010 |  | X |  |  |  |  |
| Ramezani 2011 |  |  |  |  |  | X |
| Rawal 2018 |  | X |  |  |  |  |
| Retnakaran 2017 |  | X |  |  | X |  |
| Shen 2006 |  | X |  |  |  |  |
| Shen 2018 |  |  |  |  |  | X |
| Sreelakshmi 2015 |  | X | X |  |  |  |
| Sudasinghe 2018 |  | X |  |  |  |  |
| Tam 2007 |  |  |  | X |  | X |
| Tehrani 2012 |  |  |  | X |  | X |
| Vambergue 2007 | X |  |  | X |  |  |
| Vambergue 2008 |  | X |  |  | X |  |
| Vigneault 2015 |  |  |  |  | X |  |
| Wang 2012 |  | X |  |  | X | X |
| Wender-Ozegowska 2007 |  | X |  |  |  |  |
| Xiang 2011 |  |  |  |  |  | X |
| Yefet 2019 |  |  |  |  | X |  |
| **Total (No. of publications per review)** | **20** | **45** | **5** | **30** | **20** | **32** |
| **Grand Total (N)** | **152** |  |  |  |  |  |
| **Rows (r)** | **82** |  |  |  |  |  |
| **Columns (c)** | **6** |  |  |  |  |  |
| **Corrected Covered Area (CCA)** | **17.07317073** |  |  |  |  |  |

CCA = Corrected covered area. Calculation = CCA (%) = N-r/ rc-r: Where N = Number of included publications (sum of checked boxes), r = number of rows (primary studies), c = number of columns (number of systematic reviews).

### Table S5b: Hypertensive Disorders of Pregnancy and Type 2 Diabetes Mellitus

| **Systematic review** | **Wang 2017** | **Zhao 2021** |
| --- | --- | --- |
| **Overlapping associations** | **T2DM** | **T2DM** |
| **Primary stududy** |  |  |
| Wang 2012 | X | X |
| Heida 2015 | X | X |
| Kurabayashi 2013 | X |  |
| Garovic 2010 | X |  |
| Hashemi 2013 | X |  |
| Marin 2000 | X |  |
| Callaway 2007 | X | X |
| Feig 2013 |  | X |
| Lykke 2009 |  | X |
| Savitz 2014 |  | X |
| Hwu 2016 |  | X |
| Stuart 2018 |  | X |
| Timpka 2018 |  | X |
| Timpka 2019 |  | X |
| Carr 2009 |  | X |
| Engeland 2011 |  | X |
| Kuo 2018 |  | X |
| Libby 2007 |  | X |
| Mannisto 2013 |  | 1 |
| **Total (No. of publications per review)** | **7** | **15** |
| **Grand Total (N)** | **22** |  |
| **Rows (r)** | **19** |  |
| **Columns (c)** | **2** |  |
| **Corrected Covered Area (CCA)** | **15.7894737** |  |

CCA = Corrected covered area. Calculation = CCA (%) = N-r/ rc-r: Where N = Number of included publications (sum of checked boxes), r = number of rows (primary studies), c = number of columns (number of systematic reviews).

### Table S5c: Pre-eclampsia and Type 2 Diabetes Mellitus

| **Systematic review** | **Dall'Asta 2021** | **Wang 2017** | **Wu 2016** | **Zhao 2021** |
| --- | --- | --- | --- | --- |
| **Overlapping associations** | **T2DM** | **T2DM** | **T2DM** | **T2DM** |
| **Primary stududy** |  |  |  |  |
| Libby 2007 | X | X | X | X |
| Callaway 2007 | X |  |  |  |
| Lykke 2009 | X | X | X | X |
| Edlow 2009 | X |  |  |  |
| Mannisto 2013 | X | X |  | X |
| Savitz 2014 | X | X | X | X |
| Kuo 2018 | X |  |  | X |
| Stuart 2018 | X |  |  | X |
| Engeland 2011 |  | X |  | X |
| Sibai 1986 |  | X |  |  |
| Wang 2012 |  | X |  | X |
| Carr 2009 |  | X |  | X |
| Feig 2013 |  | X |  | X |
| Magnussen 2009 |  | X |  |  |
| Hashemi 2012 |  |  | X |  |
| Hwu 2016 |  |  |  | X |
| **Total (No. of publications per review)** | **8** | 10 | 4 | 11 |
| **Grand Total (N)** | **33** |  |  |  |
| **Rows (r)** | **16** |  |  |  |
| **Columns (c)** | **4** |  |  |  |
| **Corrected Covered Area (CCA)** | **35.41666667** |  |  |  |

CCA = Corrected covered area. Calculation = CCA (%) = N-r/ rc-r: Where N = Number of included publications (sum of checked boxes), r = number of rows (primary studies), c = number of columns (number of systematic reviews).

### Table S5d: Pre-eclampsia and Hypertension

| **Systematic review** | **Alonso-Ventura 2020** | **Bellamy 2007** | **Brown 2013** | **Dall'Asta 2021** | **Xu 2022** |
| --- | --- | --- | --- | --- | --- |
| **Overlapping associations** | **Hypertension** | **Hypertension** | **Hypertension** | **Hypertension** | **Hypertension** |
| **Primary study** |  |  |  |  |  |
| Adams 1961 |  | X | X |  |  |
| Auger 2017 |  |  |  |  |  |
| Aukes 2009 |  |  | X |  |  |
| Aykas 2015 | X |  |  |  |  |
| Behrens 2017 |  |  |  | X |  |
| Berends 2008 |  |  | X |  |  |
| Best 2017 |  |  |  | X |  |
| Bhattacharya 2012 |  |  |  | X |  |
| Blaauw 2006 |  |  | X |  |  |
| Black 2016 |  |  |  | X |  |
| Bokslag 2017 | X |  |  |  |  |
| Callaway 2011 |  |  |  | X |  |
| Canti 2010 |  |  | X |  |  |
| Carleton 1988 | X | X | X |  |  |
| Chambers 2001 | X |  |  |  |  |
| Christensen 2016 | X |  |  |  |  |
| Diehl 2008 |  |  | X |  |  |
| Drost 2012 | X |  |  |  | X |
| Edlow 2009 |  |  | X | X | X |
| Egeland 2018 |  |  |  | X |  |
| Epstein 1964 |  | X | X |  |  |
| Garovic 2017 | X |  |  |  |  |
| Gaugler-Senden 2008 |  |  | X |  |  |
| Grandi 2015 |  |  |  | X |  |
| Haas 2019 |  |  |  | X |  |
| Hannaford 1997 |  | X | X |  |  |
| Haukkamaa 2009 |  |  | X |  |  |
| Hermes 2013 |  |  |  | X |  |
| Hubel 2000 |  | X | X |  |  |
| Kaaja 2005 |  |  | X |  |  |
| Kharazmi 2007 |  |  | X |  |  |
| Laivuori 1996 |  | X | X |  |  |
| Leon 2019 |  |  |  | X |  |
| Lykke 2009 |  |  | X |  |  |
| Magnussesn 2009 |  |  | X |  |  |
| Mangos 2012 | X |  |  |  |  |
| Mannisto 2013 |  |  |  | X |  |
| Manten 2007 |  |  | X |  |  |
| Marin 2000 |  | X | X |  |  |
| McDonald 2013 | X |  |  |  |  |
| Melchiorre 2011 |  |  | X |  |  |
| Nisell 1995 |  | X | X |  |  |
| North 1996 |  | X | X |  |  |
| Ostlund 2013 | X |  |  |  |  |
| Portelinha 2010 |  |  | X |  |  |
| Sattar 2003 | X | X | X |  |  |
| Shahbazian 2011 |  |  | X |  |  |
| Shammas 2000 |  | X | X |  | X |
| Sibai 1986 |  | X | X |  |  |
| Sibai 1991 |  |  |  |  |  |
| Spaan 2009 |  |  | X |  |  |
| Stuart 2018 |  |  |  | X |  |
| Tooher 2017 |  |  |  | X |  |
| van Oostwaard 2012 |  |  |  |  |  |
| van Oostwaard 2014 |  |  |  |  |  |
| Wilson 2003 |  | X | X |  | X |
| Yeh 2014 |  |  |  | X |  |
| Zhang 2015 |  |  |  |  |  |
| Zoet 2018 | X |  |  |  |  |
| Ghossein-Doha 2014 |  |  |  |  | X |
| White 2016 |  |  |  |  | X |
| Shahul 2018 |  |  |  |  | X |
| Kuo 2018 |  |  |  |  | X |
| Gastrich 2019 |  |  |  |  | X |
| Gimenez 2020 |  |  |  |  | X |
| Watanabe 2020 |  |  |  |  | X |
| deMartely 2021 |  |  |  |  | X |
| Garovic 2020 |  |  |  |  | X |
| **Total (No. of publications per review)** | **12** | **13** | **30** | **15** | **13** |
| **Grand Total (N)** | **83** |  |  |  |  |
| **Rows (r)** | **68** |  |  |  |  |
| **Columns (c)** | **5** |  |  |  |  |
| **Corrected Covered Area (CCA)** | **5.514705882** |  |  |  |  |

### Table S5e: Hypertensive Disorders of Pregnancy and Hypertension

| **Systematic review** | **Giorgione 2021** | **Wu 2020** | **Sukmanee 2022** | **Xu 2022** |
| --- | --- | --- | --- | --- |
| **Overlapping associations** | **Hypertension** | **Hypertension** | **Hypertension** | **Hypertension** |
| **Primary study** |  |  |  |  |
| Amiri 2019 |  |  | X |  |
| Auger 2017 |  | X |  |  |
| Bar 1999 | X |  |  |  |
| Berends 2008 |  | X |  |  |
| Bergen 2018 |  |  | X |  |
| Best 2017 |  | X | X |  |
| Bhattacharya 2012 |  | X |  |  |
| Blaauw 2006 |  | X |  |  |
| Black 2016 | X |  |  |  |
| Bokslag 2018 |  |  | X |  |
| Breetveld 2015 |  |  | X |  |
| Callaway 2013 |  |  | X |  |
| Canti 2010 |  | X |  |  |
| Collen 2013 |  |  | X |  |
| Diehl 2008 |  | X |  |  |
| Ditisheim 2017 | X |  |  |  |
| Drost 2012 |  | X | X |  |
| Dunietz 2017 |  |  | X |  |
| Edlow 2009 | X | X | X |  |
| Egeland 2018 |  |  | X |  |
| Ehrenthal 2014 | X |  |  | X |
| Garovic 2010 |  | X | X | X |
| Garovic 2020 |  |  | X | X |
| Gaugler-Senden 2008 |  | X |  |  |
| Ghossein-Doha 2014 |  |  | X |  |
| Ghossein-Doha 2017 |  |  | X |  |
| Grandi 2017 |  |  | X |  |
| Grandi 2018 |  | X |  |  |
| Haas 2019 |  |  | X |  |
| Hannaford 1997 |  | X |  |  |
| Haukkamaa 2009 |  | X |  |  |
| Hauspurg 2019 | X |  |  |  |
| Heid 2015 |  | X |  |  |
| Honigberg 2019 |  |  | X | X |
| Huang 2020 |  |  | X |  |
| Hubel 2000 |  | X |  |  |
| Kaaja 2005 |  | X |  |  |
| Kennedy 2022 |  |  | X |  |
| Kharazmi 2007 |  | X |  |  |
| Kuo 2018 |  | X |  |  |
| Kurabayashia 2013 |  | X |  |  |
| Kvehaugen 2014 |  |  | X |  |
| Leon 2019 |  | X |  |  |
| Li 2018 |  |  | X |  |
| Linderberg 2016 |  |  |  | X |
| Lykke 2009 |  | X | X |  |
| Magnussen 2009 |  | X |  |  |
| Mannisto 2013 |  | X |  |  |
| Manten 2007 |  | X |  |  |
| Marin 2000 |  | X |  | X |
| Melchiorre 2011 | X | X | X |  |
| Mito 2018 |  | X |  | X |
| Moe 2019 | X |  |  |  |
| Moe 2020 |  |  | X |  |
| Mooij 2021 |  |  | X |  |
| Moreira 2009 |  |  |  | X |
| Ntlemo 2021 |  |  | X |  |
| Nuckols 2021 |  |  | X |  |
| Osoti 2019 | X |  | X |  |
| Ostlund 2013 |  |  | X |  |
| Perez-Adan |  |  | X |  |
| Portelinha 2010 |  | X |  |  |
| Qasim 2016 |  |  |  | X |
| Redman 2019 | X |  |  |  |
| Sattar 2003 |  | X |  |  |
| Shahbazian 2011 |  | X |  |  |
| Shahul 2018 | X |  |  | X |
| Shalom 2013 |  |  | X |  |
| Smith 2019 | X |  | X |  |
| Spaan 2009 |  | X |  |  |
| Spaan 2012 | X |  |  |  |
| Stuart 2018 |  | X |  |  |
| Timpka 2017 |  |  | X |  |
| van Rijn 2013 | X |  |  |  |
| Wagata 2020 |  |  | X |  |
| Watanabe 2020 |  |  |  | X |
| Weissgerber 2016 |  | X |  |  |
| Wilson 2003 |  | X |  |  |
| Zhou 2014 |  |  | X |  |
| **Total (No. of publications per review)** | **14** | **35** | **36** | **11** |
| **Grand Total (N)** | **96** |  |  |  |
| **Rows (r)** | **79** |  |  |  |
| **Columns (c)** | **4** |  |  |  |
| **Corrected Covered Area (CCA)** | **7.172995781** |  |  |  |

### Table S5f: Preeclampsia and Diastolic blood pressure

| **Systematic review** | **Alonso-Ventura 2020** | **Cavalli 2021** |
| --- | --- | --- |
| **Overlapping associations** | **DBP** | **DBP** |
| **Primary study** |  |  |
| Akhter 2013 | x |  |
| Andersgaard 2012 | x |  |
| Aykas 2015 | x |  |
| Bar 1999 | x |  |
| Barden 1999 | x |  |
| Berends 2008 | x |  |
| Bhasin 2013 |  | x |
| Bokslag 2017 | x |  |
| Breetveld 2015 | x | x |
| Canti 2010 |  | x |
| Carleton 1988 | x |  |
| Chambers 2001 | x |  |
| Christensen 2016 | x |  |
| Coffeng 2010 | x |  |
| Dantas 2013 | x |  |
| Drost 2012 | x |  |
| Fraser 2012 |  | x |
| Freeman 2004 | x |  |
| Garovic 2017 | x |  |
| Girouard 2007 | x |  |
| He 1999 | x |  |
| Honigberg 2019 |  | x |
| Hubel 2008 | x |  |
| Innes 2005 | x |  |
| Kvehaugen 2010 | x |  |
| Laivuori 1996 | x |  |
| Lampinen 2008 | x |  |
| Mangos 2012 | x |  |
| Manten 2007 | x |  |
| McDonald 2013 | x |  |
| Miller 2016 |  | x |
| Nisell 1999 | x |  |
| Nohira 2013 | x |  |
| Ostlund 2013 | x |  |
| Portelinha 2010 | x |  |
| Pouta 2004 | x |  |
| Romundstad 2010 | x |  |
| Sattar 2003 | x |  |
| Smith 2009 | x |  |
| Soma-Pillay 2018 |  | x |
| Spaan 2010 | x |  |
| Suzuki 2008 | x |  |
| Timpka 2016 |  | x |
| White 2016 |  | x |
| Zoet 2018 | x |  |
| **Total (No. of publications per review)** | **37** | **9** |
| **Grand Total (N)** | **46** |  |
| **Rows (r)** | **45** |  |
| **Columns (c)** | **2** |  |
| **Corrected Covered Area (CCA)** | **2.22222222** |  |

### Table S5g: Preeclampsia and systolic blood pressure

| **Systematic review** | **Alonso-Ventura 2020** | **Cavalli 2021** |
| --- | --- | --- |
| **Overlapping associations** | **SBP** | **SBP** |
| **Primary study** |  |  |
| Akhter 2013 | x |  |
| Andersgaard 2012 | x |  |
| Aykas 2015 | x |  |
| Bar 1999 | x |  |
| Barden 1999 | x |  |
| Berends 2008 | x |  |
| Bhasin 2013 |  | x |
| Bokslag 2017 | x |  |
| Breetveld 2015 | x | x |
| Canti 2010 |  | x |
| Carleton 1988 | x |  |
| Chambers 2001 | x |  |
| Christensen 2016 | x |  |
| Coffeng 2010 | x |  |
| Dantas 2013 | x |  |
| Drost 2012 | x |  |
| Fraser 2012 |  | x |
| Freeman 2004 | x |  |
| Garovic 2017 | x |  |
| Girouard 2007 | x |  |
| Hamad 2007 | x |  |
| He 1999 | x |  |
| Honigberg 2019 |  | x |
| Hubel 2008 | x |  |
| Innes 2005 | x |  |
| Kvehaugen 2010 | x |  |
| Laivuori 1996 | x |  |
| Lampinen 2008 | x |  |
| Mangos 2012 | x |  |
| Manten 2007 | x |  |
| McDonald 2013 | x |  |
| Miller 2016 |  | x |
| Nisell 1999 | x |  |
| Nohira 2013 | x |  |
| Ostlund 2013 | x |  |
| Portelinha 2010 | x |  |
| Pouta 2004 | x |  |
| Romundstad 2010 | x |  |
| Sattar 2003 | x |  |
| Smith 2009 | x |  |
| Soma-Pillay 2018 |  | x |
| Spaan 2010 | x |  |
| Suzuki 2008 | x |  |
| Timpka 2016 |  | x |
| White 2016 |  | x |
| Zoet 2018 | x |  |
| **Total (No. of publications per review)** | **38** | **9** |
| **Grand Total (N)** | **47** |  |
| **Rows (r)** | **46** |  |
| **Columns (c)** | **2** |  |
| **Corrected Covered Area (CCA)** | **2.17391304** |  |

## Table S6: General characteristics of reviews with overlapping associations.

| **Index of overlapping associations** | **Study ID** | **AMSTAR 2 rating** | **Reproductive factor** | **Outcome** | **Synthesis type (number)** | **Corrected covered area (CCA)** | **Cochrane or non-Cochrane** | **Decision to retain** |
| --- | --- | --- | --- | --- | --- | --- | --- | --- |
|  |  |  |  |  |  |  |  | **✓= Yes 🗶= No** |
| 1 | Bellamy 2009 | Low | GDM | T2DM | MA (20) | 15.08% very high | non-Cochrane | **🗶** |
|  | Dennison 2021 | Low | GDM | T2DM | MA (45) |  | non-Cochrane | **🗶** |
|  | Gadve 2021 | High | GDM | T2DM | MA (5) |  | non-Cochrane | **🗶** |
|  | Song 2018 | Low | GDM | T2DM | MA (17) |  | non-Cochrane | **🗶** |
|  | Vounzoulaki 2020 | High | GDM | T2DM | MA (20) |  | non-Cochrane | ✓ |
|  | You 2021 | Low | GDM | T2DM | MA (39) |  | non-Cochrane | **🗶** |
| 3 | Wang 2017 | High | HDP | T2DM | MA (7) | 15.7% very high | non-Cochrane | **🗶** |
|  | Zhao 2021 | High | HDP | T2DM | MA (15) |  | non-Cochrane | ✓ |
| 4 | Dall'Asta 2021 | Moderate | Pre-eclampsia | T2DM | MA (8) | 27.8% very high | non-Cochrane | **🗶** |
|  | Wang 2017 | High | Pre-eclampsia | T2DM | MA (10) |  | non-Cochrane | **🗶** |
|  | Wu 2016 | Low | Pre-eclampsia | T2DM | MA (4) |  | non-Cochrane | **🗶** |
|  | Zhao 2021 | High | Pre-eclampsia | T2DM | MA (11) |  | non-Cochrane | ✓ |

GDM – Gestational diabetes mellitus, HDP – Hypertensive disorders of pregnancy, T2DM – Type 2 diabetes mellitus, MA – Meta-analysis

## Table S7: List of reviews with overlapping associations excluded from analysis

| **1st Author and Year** | **Title** |
| --- | --- |
| Dennison 2021 | The absolute and relative risk of type 2 diabetes after gestational diabetes: A systematic review and meta-analysis of 129 studies. |
| Gadve 2021 | Risk of Developing Type 2 Diabetes Mellitus in South Asian Women with History of Gestational Diabetes Mellitus: A Systematic Review and Meta-Analysis. |
| Li 2020 | Incidence Rate of Type 2 Diabetes Mellitus after Gestational Diabetes Mellitus: A Systematic Review and Meta-Analysis of 170,139 Women. |
| Song 2018 | Long-term risk of diabetes in women at varying durations after gestational diabetes: a systematic review and meta-analysis with more than 2 million women |
| Wang 2017 | Hypertensive disorders during pregnancy and risk of type 2 diabetes in later life: a systematic review and meta-analysis |
| Wu 2016 | Pre-eclampsia is associated with a twofold increase in diabetes: a systematic review and meta-analysis |
| You 2021 | Risk of type 2 diabetes mellitus after gestational diabetes mellitus: A systematic review & meta-analysis |

## Table S8: Exposure definitions and extend of risk factors adjustment in systematic reviews included in the umbrella review

| **Author/Year** | **Definition of pregnancy complications** | **Risk factors factors adjusted in included primary studies** |
| --- | --- | --- |
| Alonso-Ventura 2020 | **Pre-eclampsia:** New onset hypertension and proteinuria appearing after 20 weeks of pregnancy according to different scientific societies such as the American College of Obstetrics and Gynaecologists (ACOG), the International Society for the study of Hypertension in Pregnancy (ISSHP), the National High Blood Pressure Education Program Working Group (NHBPEPWG), the World Health Organization International Classification of Diseases (ICD) | **The review only provided information matching variables for controls.**  gestational age, age, parity, time of delivery, ethnicity, weight, BMI,  smoking habits and family history of DM, CVD and preeclampsia,  contraceptive intake, alcohol consumption  NB: Eleven studies did not match for controls |
| Bellamy 2007 | **Pre-eclampsia** was normally defined as the onset of a blood pressure level exceeding 140/90 mm Hg with proteinuria greater than 0.3 g/24 h after 20 weeks of gestation  NB: One study used diagnosis of pre-eclampsia in medical records to define exposure | BMI, smoking, socioeconomic status, hypercholesterolaemia, type 2 diabetes  **NB:** Seven primary studies did not adjust for any confounders |
| Brown 2013 | **Pre-eclampsia** was explicitly defined as hypertension (BP exceeding 140/90 mmHg) in combination with proteinuria (0.3 g/24 h) after 20 weeks gestation | Not provided |
| Dall'Asta 2021 | **Pre-eclampsia** was defined as new-onset hypertension (>=140 mmHg systolic or‚<=90 mmHg diastolic) at or after 20 weeks of gestation in combination with the appearance of proteinuria (>0.3 g/24 h) at or after 20 weeks of gestation | Not provided. Authors provided adjusted estimates, but no information was provided on variables adjusted for. |
| Giorgione 2021 | Not provided | Not provided. However, the systematic review compares population statistics between controls and exposed groups by maternal age, ethnicity (black ethnicity), BMI and smoking |
| Pathirana 2021 | No restriction on definition of exposures | Common confounders adjusted for were BMI, parity, age, history of diabetes, other pregnancy complications |
| Sukmanee 2022 | **Gestational hypertension** was defined as ‚ systolic blood pressure of 140mm Hg or more or a diastolic blood pressure of 90mm Hg or more, or both detected after 20 weeks of gestation in a woman with a previously normal blood pressure. **Preeclampsia** was defined as gestational hypertension with proteinuria that severe features include systolic blood pressure of 160mm Hg or more, or diastolic blood pressure of 110mm Hg or more, thrombocytopenia, impaired liver function, renal insufficiency, pulmonary edema, new-onset headache unresponsive to medication, and visual disturbances. | Age, parity, year of delivery, race/ethnicity, GDM, smoking, caesarean section, type of insurance used for the index pregnancy, preterm delivery, SGA, placental abruption, stillbirth, DM subsequent to the index pregnancy, BMI, parity, chronic hypertension, family history of cardiovascular disease, education, DM, hypertension, dyslipidaemia, IUGR child, gestational age at echocardiography, Childbearing age, blood glucose, total cholesterol, triglycerides, LDL-C, HDL-C, hs-CRP, alcohol intake, number of cigarettes smoked, exercise, Prenatal, postpartum, and prenatal and postpartum difference in: BMI, blood pressure, fasting blood glucose, fasting insulin, total cholesterol, triglycerides, HDL-C, LDL-C, parity, multiple pregnancy, stillbirth, nativity, income, history of hyperlipidaemia, migraine, and lupus, GDM, drug use, infant sex,multiple gestation at first pregnancy, depression, dyslipidaemia, venous thromboembolism, polycystic ovary syndrome, renal disease, family history of hypertension and cardiovascular disease, number of distinct drug classes prescribed, use of statin, aspirin, anti-depressant medications, NSAIDs, oral contraceptives, and anti-migraine medications, menopausal status, history of GDM, physical activity, DASH diet, sodium/potassium intake, Systolic blood pressure, length of follow-up, comorbidities (DM, hypertension, dyslipidaemia, coronary artery disease, chronic kidney disease, COPD, cerebrovascular disease), Age at intake, visit interval, subsequent pregnancies between index and follow-up, child’s sex, marital status, mid-pregnancy poor diet quality, total energy intake, family history of coronary heart disease, hyperlipidaemia, breastfeeding, marital and employment status, median household income, chronic kidney disease, pre-existing diabetes, obesity, drug abuse, number of pregnancies, age at start of follow-up, self-reported ancestry, maternal weight at first antenatal visit, socioeconomic status, primipara, aspartate aminotransferase levels; alanine aminotransferase levels; total cholesterol levels, hospital level, living area, all comorbidities, physiological measures (SBP, DBP, fat mass percentage) and interpregnancy factors of BMI change, inter-pregnancy interval and breastfeeding duration  NB: **Ten** primary studies did not adjust for any confounders.  Common confounders were age, ethnicity, socioeconomic status, time of delivery, smoking status, BMI, family history of cardiovascular diseases, parity, history of diabetes mellitus and maternal education |
| Vounzoulaki 2020 | GDM was defined as follows.  ADA (2004)—two or more raised values during oral glucose tolerance test: fasting plasma glucose ≥95 mg/dL (5.3 mmol/L), one hour glucose ≥180 mg/dL (10.0 mmol/L), two-hour glucose ≥155 mg/dL (8.6 mmol/L), three-hour glucose ≥140 mg/dL (7.8 mmol/L) after 100 g oral glucose tolerance test  ADIPS (1998)—fasting plasma glucose ≥5.5mmol/L or two-hour glucose ≥8.0 mmol/L after 75 g oral glucose tolerance test  Carpenter and Coustan—two or more raised values during oral glucose tolerance test: fasting plasma glucose ≥95 mg/dL (5.3 mmol/L), one hour glucose ≥180 mg/dL (10.0 mmol/L), two-hour glucose ≥155 mg/dL (8.6 mmol/L), three-hour glucose ≥140 mg/dL (7.8 mmol/L) after 100 g oral glucose tolerance test  Contemporary criteria—one or more raised values during oral glucose tolerance test: fasting plasma glucose >4.8 mmol/L, one hour glucose >10.0 mmol/L, two-hour glucose >8.7 mmol/L until September 2001; since September 2001 fasting plasma glucose >4.8 mmol/L, one hour glucose >11.2 mmol/L, two-hour glucose >9.9 mmol/L after 75 g oral glucose tolerance test  EASD—two-hour glucose ≥9 mmol/L after 75 g oral glucose tolerance test  IADPSG—fasting plasma glucose ≥92 mg/dL (5.1 mmol/L), one hour glucose ≥180 mg/dL (10.0 mmol/L), two-hour glucose ≥153 mg/dL (8.5 mmol/L) after 75 g oral glucose tolerance test  NICE - patients were classified as having GDM or T2DM based on clinical codes used in the  Health Improvement Network database that was based on the classification made by NICE 2008 and 2015 guidelines for diabetes in pregnancy:  NICE 2008—fasting plasma glucose ≥7.0 mmol/L or two-hour glucose ≥7.8 mmol/L after 75 g oral glucose tolerance test  NICE 2015: fasting plasma glucose ≥5.6 mmol/L or two-hour glucose ≥7.8 mmol/L after 75 g oral glucose tolerance test  NDDG (1979)—fasting plasma glucose ≥105 mg/dL (5.8 mmol/L), one hour glucose ≥190 mg/dL (10.6 mmol/L), two-hour glucose ≥165 mg/dL (9.2  mmol/L), three-hour glucose ≥145 mg/dL (8.0 mmol/L) after 100 g oral glucose tolerance test  Second and third workshop conference on GDM—two or more raised values during oral glucose tolerance test: fasting plasma glucose ≥5.8 mmol/L, one hour glucose ≥10.6 mmol/L, two-hour glucose ≥9.2 mmol/L, three-hour glucose ≥8.1 mmol/L after 100 g oral glucose tolerance test  WHO (1985)—fasting plasma glucose ≥7.0 mmol/L or two-hour glucose ≥11.1 mmol/L after 75 g oral glucose tolerance test  WHO (1999)—fasting plasma glucose ≥7.0 mmol/L, two-hour glucose ≥7.8 mmol/L after 75 g oral glucose tolerance test | OGTT values during pregnancy, insulin treatment, maternal age, parity, year of delivery, length of follow-up, family history of diabetes, BMI at follow-up, socioeconomic status, smoking, history of GDM in a previous pregnancy, treatment with insulin during index pregnancy, birth weight, gestational age at delivery, physical activity, waist circumference, ethnicity, height, educational level, income level, systolic blood pressure, total cholesterol, triglycerides. HDL-cholesterol, comorbidity, breastfeeding, baseline b-cell function, weight, the number of previous pregnancies, the number of previous births, fasting and 1 h OGTT results and the number of glucose charts for each woman.  **NB:** There were 3 primary studies that did not adjust for any confounders.  Common confounders across the studies were maternal age, body mass index, family history of T2DM, parity, ethnicity, and socioeconomic status. |
| Xu 2022 | -Gestational hypertension - Hypertension occurring after 20 weeks of pregnancy, systolic blood pressure140 mmHg and (or) diastolic bloodpressure90 mmHg, and a return to normal blood pressure within 12 weeks after delivery; urinary protein (‚Äì); the diagnosis can be made after delivery.  -Preeclampsia - Systolic blood pressure140 mmHg and (or) diastolic blood pressure90 mmHg after 20 weeks of pregnancy, accompanied by urinary protein0.3 g/24 h, or random urinary protein (+)Or without proteinuria, but combined with any of the following: Thrombocytopenia (platelets<100)109=L)Liver function impairment (serum transaminase level is more than twice the normal value)Renal function impairment (serum creatinine level>1.1 mg/dl or more than twice the normal value)Pulmonary edemaNew central nervous system abnormalities or visual impairment  -Eclampsia - Convulsions that cannot be explained by other reasons occurring on the basis of preeclampsia.  -Preeclampsia superimposed on chronic hypertension-There was no proteinuria before pregnancy, and proteinuria was present after 20 weeks of pregnancy in women withchronic hypertension; or proteinuria was present before pregnancy, and proteinuria increased significantly after pregnancy; or blood pressure rises further; or thrombocytopenia<100l09=L; or other serious manifestations such as liver and kidney function damage, pulmonary edema, nervous system abnormalities, or visual impairment.  -Pregnancy complicated with chronichypertension - Systolic blood pressure140 mmHg and (or) diastolic blood pressure90 mmHg before 20 weeks of pregnancy (excluding trophoblastic diseases), and there was no significant aggravation during pregnancy; or hypertension was first diagnosed after 20 weeks of pregnancy and continued beyond 12 weeks postpartum. | A list of confounders adjusted in each primary study is not provided, however authors mention some studies adjusted for age and BMI at recruitment, pre-pregnancy BMI, age at first delivery and other factors. |
| Zhao 2021 | **Hypertensive disorders of pregnancy** were determined using administrative data (registries, remuneration claims, insurance databases) in nine studies, self-report in five studies, medical records in two studies and consultant obste-tricians in one study. One self-reported definition was validated by medical records and another medical record definition was validated by obstetricians.  For the studies that did include GDM, only one study specified that GDM was diagnosed by Carpenter and Coustan criteria.  In the remaining studies, GDM was ascertained in the same way as HDP, using International Classification of Diseases (ICD) diagnostic codes or self-report. | GDM, maternal age, parity, family income, smoking history, physical activity, pre-pregnancy and current BMI, chronic HTN, primigravity, PCOS, parity, socioeconomic status, hypercholesterolaemia, CVD, oral glucose-lowering drugs or insulin, prior stroke, current alcohol consumption, renal disease, urbanisation level, year of pregnancy, dyslipidaemia, date of delivery , infant birthweight, preterm delivery, small for gestational age, placental abruption, stillbirth, race/ethnicity, insurance status, maternal education, family history of chronic HTN or T2DM,diet, oral contraceptive use, year of birth, family history of DM and CVD, OGTT at age 50, menopausal status, occupation, obesity, hyperlipidaemia  **NB:** Common confounders across the studies were maternal age, body mass index, socioeconomic status, smoking status, pre-pregnancy and current BMI, parity, ethnicity and time of delivery |

## Table S8a: Extend of confounders adjustment in Alonso-Ventura 2020 review

| **Study** | **Factors** |
| --- | --- |
| Akhter T, 2013 | Gestational age |
| Andersgaard AB, 2012 | Age, Parity |
| Aykas F, 2015 | Age |
| Bar J, 1999 |  |
| Barden AE, 1999 | Age, gestational weeks |
| Berends AL, 2008 |  |
| Bokslag A, 2017 | Age, year of delivery |
| Breetveld NM, 2015 |  |
| Carleton H, 1988 | Age, year of delivery, ethnicity, weight |
| Chambers JC, 2001 |  |
| Christensen M, 2016 | Age, time since delivery |
| Coffeng SM, 2011 |  |
| Dantas EMM, 2013 |  |
| Drost JT, 2012 | Age |
| Forest JC, 2005 | Age, year of delivery |
| Freeman DJ, 2004 | Age, Parity, BMI |
| Garovic VD, 2017 | Age, Parity |
| Girouard D J, 2007 | Age, year of delivery |
| Hamad RR, 2007 | Age, Parity |
| He S, 1999 | Age, Parity, year of delivery |
| Hubel CA, 2008 | Age, date of birth |
| Innes KE, 2005 | Age, ethnicity |
| Kvehaugen AS, 2010 |  |
| Laivuori H, 1996 | Age |
| Lampinen KH, 2008 |  |
| Mangos GJ, 2012 | BMI |
| Manten GTR, 2007 |  |
| McDonald SD, 2013 | Age, child's age |
| Nisell H, 1999 | Age, year of delivery |
| Nohira T, 2013 | Age, parity, BMI, smoking status, family history of diabetes, CVD, preeclampsia |
| Östlund E, 2013 | Age, Parity, year of delivery |
| Portelinha A, 2008 | Age, BMI |
| Portelinha A, 2010 | Age |
| Pouta A, 2004 |  |
| Romundstad PR, 2010 |  |
| Sattar N, 2003 | time of pregnancy, smoking status, BMI |
| Smith GN, 2009 | Age, Parity, ethnicity |
| Spaan JJ, 2010 | Age, BMI, year of delivery |
| Suzuki H, 2008 |  |
| White WM, 2016 | Age, Parity |
| Zoet GA, 2018 | Age, ethnicity |

## Table S8b: Extend of confounders adjustment in Bellamy 2007 review

| **Study** | **Factors** |
| --- | --- |
| Adams 1961 |  |
| Epstein 1964 |  |
| Sibai 1986 |  |
| Carleton 1988 | BMI |
| Nisell 1995 |  |
| North 1996 |  |
| Laivuori 1996 |  |
| Hannaford 1997 | smoking status, socioeconomic status |
| Marin 2000 | BMI, socioeconomic status, hypercholesterolaemia, type 2 diabetes mellitus |
| Shammas 2000 |  |
| Hubel 2000 |  |
| Sattar 2003 | BMI, smoking status |
| Wilson 2003 | socioeconomic status |

## Table S8c: Extend of confounders adjustment in Pathirana 2021 review

| **Study** | **Factors** |
| --- | --- |
| Akinci 2008 | BMI, fasting and post load glucose, total cholesterol, triglyceride, HDL, LDL. |
| Anastasiou 1998 |  |
| Banerjee 2012 | BMI |
| Bently-Lewis 2015 |  |
| Bently-Lewis 2016 | age, ethnicity, gravidity, parity,SBP, BMI, GWG, birth weight, gestational age percentile,smoking status, breastfeeding at discharge, marital status, education years |
| Bo 2006 | BMI, waist circumference, blood glucose |
| Caliskan 2014 | Carotid intima medial thickness (cIMT), total cholesterol, BMI, HBA1C, and HOMA-IR |
| Carr 2006 |  |
| Celina 1983 |  |
| Charwat-Resl 2017 |  |
| Davenport 2012 |  |
| Davis 1999 (3-18 | triglycerides, BMI |
| Fakhrzadeh 2012 | BMI |
| Ferrada 2007 |  |
| Ferraz 2007 | GDM, fasting glucose. |
| Friere 2006 |  |
| Gadgil 2017 | age, weight at 40 |
| Gunderson 2010 | parity, births during interval, time to first conception, smoking, age, ethnicity |
| Gunderson 2014 |  |
| Hakkariainen 2016 |  |
| Heida 2015 | HDP, age, BMI, smoking, alcohol consumption, total cholesterol/HDL ratio |
| Hunger-Dathe 2006 |  |
| King 2009 | age, current use of estrogen, BMI |
| Ko 1999 |  |
| Krishnaveni 2007 |  |
| Lee 2008 | Ethnicity |
| Lee 2015 | age, BMI |
| Lim 2007 |  |
| Madarasz 2009 | age and BMI |
| Mai 2014 |  |
| Meier 2005 | age and BMI |
| Minoee 2017 |  |
| Moleda 2016 |  |
| Noctor 2016 | BMI |
| Noujah 2018 | gravidity, BMI |
| Rauito 2014 | age, BMI |
| Roca-Rodriguez 2014 | age, ethnicity,family history of diabetes |
| Ryan 2013 |  |
| Simmons 2017 |  |
| Sriharan 2002 | age, time from previous pregnancy, BMI, and family history of diabetes |
| Sung 2008 |  |
| Tehrani 2012 |  |
| Thomann 2008 | age, percent body fat. |
| Verma 2002 |  |
| Vilmi-Kerala 2016 |  |
| Wang 2015 |  |
| Winhofer 2014 |  |
| Zajdenverg 2014 |  |

## Table S8d: Extend of confounders adjustment in Sukmanee 2021 review

| **Author, year** | **Factors** |
| --- | --- |
| Kestenbaum, 2003 | Age, parity, year of delivery, ethnicity/ethnicity, GDM, smoking, cesarean section, type of insurance used for the index pregnancy |
| Lykke,2009 | Age at delivery, year of delivery, preterm delivery, SGA, placental abruption, stillbirth, DM subsequent to the index pregnancy |
| Edlow,2009 | ethnicity, BMI, parity, chronic hypertension |
| Garovic,2010 | ethnicity, network, family history of cardiovascular disease, education, DM, smoking, BMI, hypertension, dyslipidemia |
| Mongraw-Chaffin 2010 | IUGR child, preexisting hypertension, age, BMI, current smoking at enrollment |
| Melchiorre,2011 | Age, ethnicity, gestational age at echocardiography |
| Drost,2012 | Age, years postpartum, current smoking |
| Zhao, 2012 | Childbearing age, age at the 2006-2007 physical examination, BMI, blood pressure, fasting blood glucose, total cholesterol, triglycerides, LDL-C, HDL-C, hs-CRP |
| Collen,2013 | Age |
| Callaway,2013 | Age, ethnicity, education, alcohol intake, number of cigarettes smoked, exercise, BMI |
| Shalom,2013 |  |
| Östlund,2013 | Age, parity, date of delivery |
| Kvehaugen,2014 | Age |
| Zhou,2014 | Prenatal, postpartum, and prenatal and postpartum difference in: BMI, blood pressure, fasting blood glucose, fasting insulin, total cholesterol, triglycerides,HDL-C, LDL-C |
| Ghossein-Doha 2014 | Parity |
| Yeh, 2014 | Age, date of delivery |
| Breetveld,2014 |  |
| Ehrenthal,2015 | Age, BMI, ethnicity, family history of cardiovascular disease, tobacco use, insurance |
| Behrens,2016 | Maternal birth year, parity, multiple pregnancy, stillbirth |
| Cain, 2016 | Age, ethnicity/ethnicity, nativity, education, income, history of hyperlipidemia, migraine, and lupus, pre-pregnancy BMI, GDM, tobacco use, drug use, infant sex |
| Nelander,2016 | Age at interview, current BMI, education, current smoking |
| Pérez-Adan,2016 |  |
| Grandi,2017 | Age, smoking, BMI, excessive alcohol use, year of cohort entry, region of residence, multiple gestation at first pregnancy, depression, dyslipidemia, venous thromboembolism, polycystic ovary syndrome, GDM, DM,renal disease, migraines, family history of hypertension and cardiovascular disease, number of distinct drug classes prescribed, use of statin, aspirin, antidepressant medications, NSAIDs, oral contethnicityptives, and antimigraine medications |
| Timpka,2017 | Age, ethnicity/ethnicity, parity, BMI at age 18, updated smoking status, alcohol use, NSAIDs use, menopausal status, history of GDM, updated BMI, physical activity, DASH diet, sodium/potassium intake |
| Dunietz,2017 | ethnicity/ethnicity, pre-pregnancy BMI, age, parity, Medicaid insurance coverage at the time of the Pregnancy Outcomes and Community Health (POUCH) Study pregnancy, time between the POUCH Study pregnancy and follow-up |
| Best, 2017 | Systolic blood pressure, current age, current BMI |
| Ghossein-Doha 2017 | Parity |
| Bokslag,2018 | Age, date of delivery, smoking status, blood pressure, BMI, educational level |
| Li, 2018 | Age, ethnicity, college education, pre-pregnancy BMI, parity |
| Chen,2018 | Age and year of delivery, parity,gestational age, gestational number, length of follow-up, comorbidities (DM, hypertension, dyslipidemia, coronary artery disease, chronic kidney disease, COPD, cerebrovascular disease) |
| Bergen,2018 | Age at intake, visit interval, ethnicity, educational level, smoking, subsequent pregnancies between index and follow-up, child’s sex, BMI at follow-up |
| Theilen,2018 | 5-year age groups, year of childbirth, parity, infant sex, gestational age at delivery, parental education, ethnicity/ethnicity, marital status |
| Basit, 2018 | Age, parity, maternal birth year, region in which the child was delivered |
| Kuo, 2018 | Age, date of delivery |
| Egeland,2018 | Age at delivery, age, BMI,educational level, physical activity, daily smoking, alcohol consumption frequency, duration of lifetime oral contethnicityptive use, mid-pregnancy poor diet quality, total energy intake, multiple birth pregnancies at delivery |
| Haas, 2019 | Age, BMI, type of health insurance at Nulliparous,Pregnancy Outcomes,Study—Monitoring Mothers-tobe Heart Health Study (nuMoM2b) enrollment, ethnicity/ethnicity, smoking during the 3 months before pregnancy |
| Haug,2019 | Age, maternal birth year, highest educational level, ever daily smoking, parity before age 40 years, family history of coronary heart disease |
| Amiri,209 |  |
| Smith,2019 | Age |
| Honigberg,2019 | Age, ethnicity, BMI, ever-smoking, prevalent hypertension, hyperlipidemia, and DM |
| Ernawati,2019 |  |
| Osoti,2019 | Age, level of education, BMI, hormonal contethnicityption, breastfeeding, marital and employment status |
| Leon, 2019 | Ethnicity, age, pre-pregnancy diabetes and hypertension, index of multiple deprivation, cluster term |

## Table S8e: Extend of confounders adjustment in Vounzoulaki 2021 review

| Study | **Factors** |
| --- | --- |
| Aberg et al., 2002 | OGTT values during pregnancy, insulin treatment, maternal age, parity, year of delivery |
| Albareda et al., 2003 | age, length of follow-up, family history of diabetes, BMI |
| Aziz et al., 2018 |  |
| Chodick et al., 2010 | age, parity, BMI, socioeconomic status, smoking |
| Daly et al., 2018 | age, deprivation, BMI, smoking |
| Herath et al., 2017 | age, family history of T2DM in a first degree relative, history of GDM in a previous pregnancy, treatment with insulin during index pregnancy, birth weight, gestational age at delivery, parity |
| Huopio et al., 2014 | age, BMI, parity, follow-up time, smoking, physical activity |
| Krishnaveni et al., 2007 | age, parity, socio-economic status, family history of diabetes, waist circumference |
| A.J. Lee et al., 2007 | age, ethnicity, height, parity, BMI, birth weight, gestational age, insulin use in pregnancy, family history of diabetes |
| H. Lee et al., 2008 | age, family history of diabetes, educational level, income level, smoking, drinking status, waist circumference, systolic blood pressure, total cholesterol, triglycerides. HDL- cholesterol |
| Linne et al., 2002 |  |
| Madarasz et al., 2009 | age, BMI |
| Minooee et al., 2017 | age, BMI, family history of diabetes |
| Mukerji et al., 2012 | age, socioeconomic status, comorbidity |
| Pintaudi et al., 2015 | age |
| Retnakaran et al., 2010 | age, ethnicity, family history of diabetes, breastfeeding, baseline bcell function, waist circumference, weight |
| Vambergue et al., 2008 | BMI, age, family history of diabetes, socioeconomic status, ethnicity, OGTT values during pregnancy, insulin treatment during pregnancy. |
| Vigneault et al., 2015 |  |
| Wang et al., 2012 | age, smoking, income, BMI, systolic blood pressure, parity,ethnicity |
| Yefet et al., 2019 | age, BMI, number of previous pregnancies, number of previous births, fasting and 1 h OGTT results, number of glucose charts for each woman |

## Table S8f: Extend of confounders adjustment in Zhao 2021 review

| **Study** | **Factors** |
| --- | --- |
| Callaway et al (2007) | GDM, maternal age, parity, family income, smoking history, physical activity, BMI |
| Carr et al (2009) | GDM (stratified), chronic hypertension, age, primigravidity |
| Engeland et al (2011) | GDM (stratified), chronic hypertension, PCOS, maternal age, parity |
| Feig et al (2013) | GDM (stratified), chronic hypertension, age, socioeconomic status, hypercholesterolaemia, CVD, parity |
| Groenhof et al (2019) | BMI, smoking, oral glucose-lowering drugs or insulin |
| Heida et al (2015) | GDM (stratified), chronic hypertension, CVD, prior stroke, age, BMI, current smoking, alcohol consumption |
| Hwu et al (2016) | GDM (stratified), chronic hypertension, age, renal disease, urbanisation level; matched by age and year of pregnancy |
| Kuo et al (2018) | chronic hypertension, age, dyslipidaemia, CVD, prior stroke; matched for age and date of delivery |
| Libby et al (2007) | age, infant birthweight, socioeconomic statu |
| Lykke et al (2009) | CVD, prior stroke, preterm delivery, small for gestational age, placental abruption, stillbirth |
| Mannisto et al (2013) | GDM, chronic hypertension (stratified),BMI, smoking, parity, DM before or during pregnancy, socioeconomic status |
| Savitz et al (2014) | GDM (stratified), chronic hypertension, dyslipidaemia, CVD, prior stroke, maternal age, parity, ethnicity, insurance status, maternal education, prenatal smoking,pre-pregnancy weight, calendar time |
| Stuart et al (2018) | chronic hypertension, dyslipidaemia, CVD, prior stroke, maternal age, ethnicity, parental education, physical activity, family history of chronic hypertension or T2DM, BMI, alcohol consumption, diet, smoking history, oral contethnicityptive use |
| Timpka et al (2018) | GDM, CVD, prior stroke, year of birth, smoking habits, family history of DMand CVD, education level, BMI, OGTT at age 50 |
| Timpka et al (2019) | GDM, age, ethnicity, parity, BMI, menopausal status, parental history of diabetes, DASH diet, physical activity, alcohol intake, smoking |
| Wang et al (2012) | GDM, chronic hypertension, dyslipidaemia, age, occupation, obesity, hyperlipidaemia |

## FigS1: Word cloud of confounders adjustment reported by reviews.


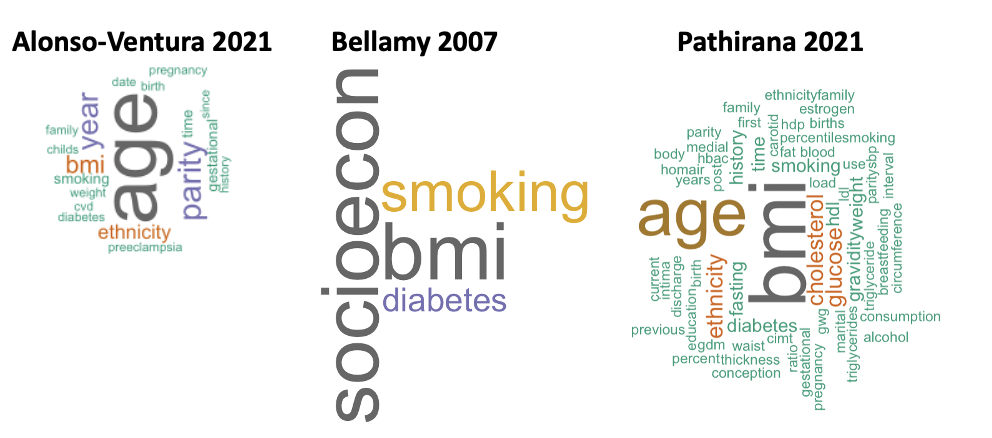


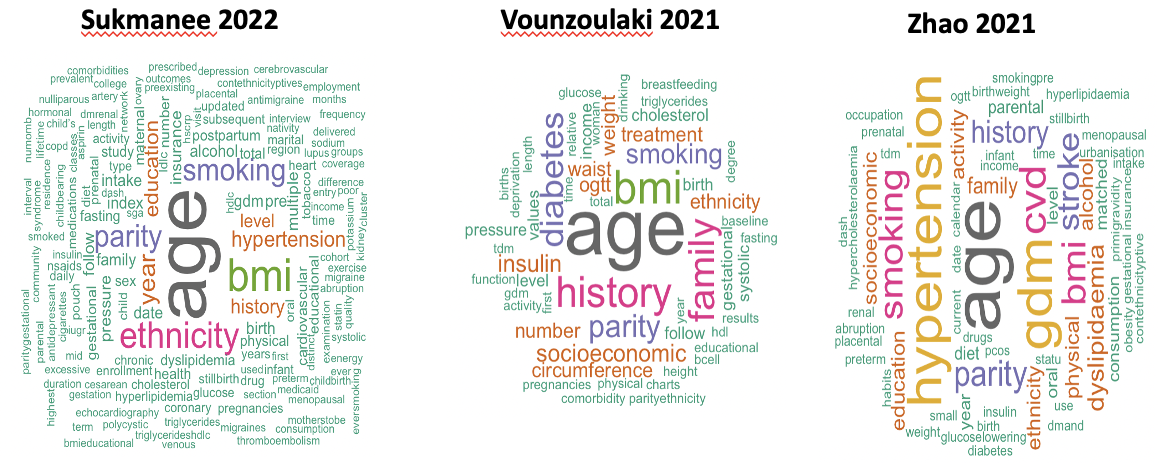


## Table S9: Tabular presentation of findings: Narrative syntheses

| **Pregnancy complication** | **Author year** | **Outcome** | **No of participants** | **Narrative summary** | **Reviewer’s conclusions** | **Overall AMSTAR 2 rating for review** |
| --- | --- | --- | --- | --- | --- | --- |
| GDM | Pathirana 2021 | SBP | NP | **Alberada 2004 (Median IQR)**  GDM 120 (85-180), Controls 110 (80-140), p-value (**<0.05)**  **Bently-Lewis 2015 (Mean SD)**  GDM (116±11), Controls (115±9), p-value (0.29)  **Hannemann 2002**  GDM (118 (109–144), Controls 113 (99–133), p-value (NS)  **Hu 1998 (Median IQR)**  GDM 116 (95-128), Controls 108 (97-120), p-value (0.04)  **Laurenborg 2005 (Median IQR)**  GDM 119 (111–126), Controls 120 (110–125), p-value (0.206)  **Levka 2015 (Reported in 2016, 2017)  (Median IQR)**  ***IADSPG criteria:*** GDM 110 (110-130), Controls 110 (110-120), p-value (NS)  ***WHO criteria:*** GDM 110 (110-130), Controls 110 (110-120), p-value (NS)  **Retnakaran 2010 (Median [IQR]) (Similarly reported in 2011)**  ***3 months:*** GDM 111 (105-118), Controls 108 (101–113), p-value (NR)  ***12 months:*** GDM 110 (103-119), Controls 109 (100-115), p-value (NR)  **Rukasaskul 2016 (Median [IQR])**  GDM 120 (100-155), Controls 110 (100-140), p-value (0.002)  **Todoric 2012 (Median IQR)**  GDM 106 (95–120), Controls 118 (110–125), p-value (NS)  **Ueland 2018 (Median IQR)**  GDM 120 (100, 130), Controls 110 (100, 120), p-value (NS)  **Verma 2002 (Mean SD)**  ***5 years:*** GDM 121.6 ± 10.8 (88), Controls 119.2 ± 9.7 (79), p-value (0.10)  ***6 years:*** GDM 121.8 ± 11.8 (87), Controls 117.9 ± 10.4 (79), p-value (0.03)  ***9 years:*** GDM 122.2 ± 11.9 (57), Controls 117.5 ± 13.2 (50), p-value (0.06)  **Wang 2012 (Mean SD) (adjusted for age)**  GDM 131 (0.6), Controls 128 (0.1), p-value (**< 0.001**) | Of the 12 studies with data not included in the meta-analysis, eight reported higher **SBP** in women with previous GDM compared to the control group with five studies showing statistical significance. | High |
| GDM | Pathirana 2021 | DBP | NP | **Alberada 2004 (Median IQR)**  GDM 78 (50-100), Controls 70 (50-100),p-value (**<0.05)**  **Bently-Lewis 2015 (Mean SD)**  GDM (73±8), Controls (71±7),p-value (0.09)  **Hannemann 2002**  GDM 74 (64–92), Controls 74 (53–92), p-value (NS)  **Hu 1998 (Median IQR)**  GDM 78 (55-84), Controls 68 (56-81), p-value (0.02)  **Laurenborg 2005 (Median IQR)**  GDM 73 (66–78), Controls 75 (70–80), p-value (<0.0005)  **Levka 2015 (Reported in 2016, 2017)  (Median IQR)**  ***IADSPG criteria:*** GDM 70 (65-75), Controls 70 (60-75), p-value (NS)  ***WHO criteria:*** GDM 70 (65-80), Controls 70 (65-75), p-value (NS)  **Retnakaran 2010 (Median [IQR]) (Similarly reported in 2011)**  ***3 months:*** GDM 66 [60–72], Controls 66 (60–70), p-value (NR)  ***12 months:*** GDM 66 (60–71), Controls 64 (59–70), p-value (NR)  **Rukasaskul 2016 (Median [IQR])**  GDM 70 (50-91), Controls 60 (60-80), p-value (0.092)  **Todoric 2012 (Median IQR)**  GDM 65 (60–70), Controls 73 (65–80), p-value (NS)  **Ueland 2018 (Median IQR)**  GDM 70 (65, 74), Controls 70 (65,75), p-value (NS)  **Verma 2002 (Mean SD)**  ***5 years:*** GDM 72.3 ±10.6 (88), Controls 70.8 ± 10.4 (79), p-value (0.10)  ***6 years:*** GDM 72.0 ± 11.1 (87), Controls 68.9 ± 9.3 (79), p-value (0.03)  ***9 years:*** GDM 72.4 ± 8.8 (57), Controls 69.9 ± 11.2 (50), p-value (0.20)  **Wang 2012 (Mean SD) (adjusted for age)**  GDM 76 (0.4), Controls 76 (0.1), p-value (0.2) | Of the 12 studies not included in the meta-analysis, six reported higher **DBP** in women with previous GDM com- pared to the control group, with three studies showing statistical significance | High |
| HDP | Zhao 2021 | T2DM | 2816 | **Groenhof 2019**  Overall, diabetes mellitus was more prevalent in HDP women (*P*_=_0.001). The increase of diabetes mellitus prevalence over time was steeper in women after HDP than after NP (*P*=0.008). | Women with prior HDP developed diabe- tes at earlier ages than women with normotensive pregnancies | High |

GDM: Gestational diabetes mellitus, HDP – hypertensive disorders of pregnancy, SBP – systolic blood pressure, DBP – Diastolic blood pressure

## Table S10: Tabular presentation of original estimates: Meta-analysis

| **Reproductive factor** | **Study identity Author/Year** | **Outcome** | **Overall or subgroup meta-analysis** | **Secondary Analysis** | **No of studies included** | **Participants** | **Evidence synthesis** | **I2 statistic** | **Overall evidence of publication bias** | **AMSTAR 2 rating** |
| --- | --- | --- | --- | --- | --- | --- | --- | --- | --- | --- |
| GDM | Vounzoulaki 2020 | T2DM | Overall |  | 20 | 1332373 | RR 9.51 (7.14 - 12.67) | 96.5 | P=0.58 | High |
|  | **Subgroup analysis by Ethnicity** | | | | | | | |  |  |
|  | Vounzoulaki 2020 | T2DM | Subgroup | White | 6 | 187532 | RR 16.28 (15.01 - 17.66) |  |  |  |
|  | Vounzoulaki 2020 | T2DM | Subgroup | Non-White | 4 | 2538 | RR 10.38 (4.61 - 23.39) |  |  |  |
|  | Vounzoulaki 2020 | T2DM | Subgroup | Mixed | 10 | 1142303 | RR 8.31 (5.44 - 12.69) |  |  |  |
|  | **Subgroup analysis by length of follow-up (Vounzoulaki 2020)** | | | | | | |  |  |  |
|  | Vounzoulaki 2020 | T2DM | Subgroup | 1-5 years | 6 | 1566 | RR 17.06 (8.95 - 32.55) |  |  |  |
|  | Vounzoulaki 2020 | T2DM | Subgroup | > 5 - 10 years | 7 | 223945 | RR 10.42 (5.68 - 19.11) |  |  |  |
|  | Vounzoulaki 2020 | T2DM | Subgroup | More than 10 years | 7 | 1106862 | RR 8.09 (7.14 - 12.67) |  |  |  |
| HDP | Zhao 2021 | T2DM | Overall |  | 15 | 3095457 | HR 2.24 (1.95 - 2.58) | 94 | P=0.6609 | High |
| Gestational Hypertension | Zhao 2021 | T2DM | Overall |  | 7 | NP | HR 2.19 (1.69 - 2.84) | 87 |  |  |
| Pre-eclampsia | Zhao 2021 | T2DM | Overall |  | 11 | NP | HR 2.56 (2.02 - 3.24) | 94 |  |  |
| Preterm pre-eclampsia | Zhao 2021 | T2DM | Overall |  | 3 | NP | HR 3.05 (2.05 - 4.56) | 82 |  |  |
|  | **Subgroup analysis after controlling for GDM (Zhao 2021)** | | | | | | | |  |  |
| HDP | Zhao 2021 | T2DM | Subgroup |  | 11 | NP | HR 2.01 (1.77 - 2.28) |  |  |  |
| Gestational hypertension | Zhao 2021 | T2DM | Subgroup |  | 5 | NP | HR 2.01 (1.54-2.63) |  |  |  |
| Pre-eclampsia | Zhao 2021 | T2DM | Subgroup |  | 7 | NP | HR 2.38 (1.92-2.96) |  |  |  |
|  | **Subgroup analysis after controlling for Chronic hypertension (Zhao 2021)** | | | | | | | |  |  |
| HDP | Zhao 2021 | T2DM | Subgroup |  | 9 | NP | HR 2.29 (1.91-2.74) |  |  |  |
| Gestational hypertension | Zhao 2021 | T2DM | Subgroup |  | 5 | NP | HR 2.19 (1.59-3.01) |  |  |  |
| Pre-eclampsia | Zhao 2021 | T2DM | Subgroup |  | 7 | NP | HR 3.03 (2.33-3.94) |  |  |  |
|  | **Subgroup analysis after controlling for BMI (Zhao 2021)** | | | | | | | |  |  |
| HDP | Zhao 2021 | T2DM | Subgroup |  | 8 | NP | HR 1.69 (1.47 - 1.94) |  |  |  |
| Gestational hypertension | Zhao 2021 | T2DM | Subgroup |  | 4 | NP | HR 1.57 (1.29 - 1.92) |  |  |  |
| Pre-eclampsia | Zhao 2021 | T2DM | Subgroup |  | 4 | NP | HR 2.32 (1.46 - 3.68) |  |  |  |
|  | **Subgroup analysis after controlling for CVD (Zhao 2021)** | | | | | | | |  |  |
| HDP | Zhao 2021 | T2DM | Subgroup |  | 8 | NP | HR 2.40 (2.02 - 2.85) |  |  |  |
| Gestational hypertension | Zhao 2021 | T2DM | Subgroup |  | 5 | NP | HR 2.18 (1.57 - 3.01) |  |  |  |
| Pre-eclampsia | Zhao 2021 | T2DM | Subgroup |  | 6 | NP | HR 3.10 (2.20 - 4.37) |  |  |  |
|  | **Subgroup analysis after controlling for BMI, GDM, Chronic hypertension and CVD (Zhao 2021)** | | | | | | | |  |  |
| HDP | Zhao 2021 | T2DM | Subgroup |  | 3 | NP | HR 1.89 (1.17 - 3.04) |  |  |  |
| Gestational hypertension | Zhao 2021 | T2DM | Subgroup |  | 2 | NP | NP |  |  |  |
| Pre-eclampsia | Zhao 2021 | T2DM | Subgroup |  | 2 | NP | NP |  |  |  |
|  | **Subgroup analysis for individual follow-up duration (Zhao 2021)** | | | | | | | |  |  |
| HDP | Zhao 2021 | T2DM | Subgroup | < 20 years | 7 | NP | HR 2.64 (2.23 - 3.12) |  |  |  |
|  | Zhao 2021 | T2DM | Subgroup | >= 20 years | 2 | NP | HR 1.34 (1.20 - 1.50) |  |  |  |
| Pre-eclampsia | Alonso-Ventura 2020 | DBP | Overall |  | 37 | 17232 | MD 6.79 (5.62 - 7.96) | 83 | There was no asymmetry of points in the funnel plot hence no publication bias | Moderate |
|  | Alonso-Ventura 2020 | Hypertension | Overall |  | 12 | 2261 | RD 0.24 (0.15 - 0.33) | 89 |  |  |
|  | Alonso-Ventura 2020 | SBP | Overall |  | 38 | 17267 | MD 8.28 (6.85 - 9.71) | 78 |  |  |
|  | Bellamy 2007 | Hypertension | Overall |  | 13 | 19744 | RR 3.7 (2.7 - 5.05) | 62.6 | P=0.014 | Moderate |
|  | Brown 2013 | Hypertension | Overall |  | 30 | 822555 | RR 3.13 (2.51 - 3.89) | 88.6 | P=0.854 | High |
|  | Dall'Asta 2021 | Hypertension | Overall |  | 21 | 2711443 | OR 3.93 (3.08 - 5.02) | 99 | Not assessed | Moderate |
|  | Dall'Asta 2021 | Hypertension | Overall |  | 15 | 2695024 | OR 3.74 (2.87 - 4.87) | 99 | Not assessed |  |
| HDP | Giorgione 2021 | Hypertension | Overall |  | 14 | 7580 | OR 5.75 (3.92 - 8.44) | 49 | P=0.890 | High |
|  | Giorgione 2021 | Hypertension (excluding Chronic hypertension) | Overall |  | 15 | 8041 | OR 6.28 (4.18 - 9.43) | 56 |  |  |
|  | **Subgroup analysis for individual follow-up duration (Giorgione 2021)** | | | | | | | |  |  |
|  | Giorgione 2021 | Hypertension (excluding Chronic hypertension) | Subgroup | Upto six months | 3 | 338 | OR 13.39 (1.27 - 141.04) |  |  |  |
|  | Giorgione 2021 | Hypertension (excluding Chronic hypertension) | Subgroup | Six months to a year | 3 | 6037 | OR 4.13 (2.82 - 6.07) |  |  |  |
|  | Giorgione 2021 | Hypertension (excluding Chronic hypertension) | Subgroup | One to two years | 8 | 1205 | OR 8.73 (4.66 - 16.35) |  |  |  |
| GDM | Pathirana 2021 | DBP | Overall |  | 48 | 49495 | OR MD 1.89 (1.32 - 2.46) | 83 | Visual analysis of funnel plots showed no publication bias | High |
|  | Pathirana 2021 | SBP | Overall |  | 48 | 50118 | OR MD 2.47 (1.74 - 3.4) | 79 |  |  |
|  | **Subgroup analysis for individual follow-up duration (Pathirana 2021)** | | | | | | | |  |  |
|  | Pathirana 2021 | DBP | Subgroup | < 1 year | NP | 1746 | OR MD 2.48 (0.58 - 4.37) |  |  |  |
|  | Pathirana 2021 | SBP | Subgroup | < 1 year | NP | 1826 | OR MD 3.47 (1.26 - 5.68) |  |  |  |
|  | Pathirana 2021 | DBP | Subgroup | 1-5 years | NP | 19676 | OR MD 1.37 (0.20 - 2.54) |  |  |  |
|  | Pathirana 2021 | SBP | Subgroup | 1-5 years | NP | 19701 | OR MD 2.26 (0.27 - 4.25) |  |  |  |
|  | Pathirana 2021 | DBP | Subgroup | 5-10 years | NP | 2184 | OR MD 7.17 (-1.69-16.03) |  |  |  |
|  | Pathirana 2021 | SBP | Subgroup | 5-10 years | NP | 1965 | OR MD 3.96 (2.36 - 5.56) |  |  |  |
|  | Pathirana 2021 | DBP | Subgroup | > 10 years | NP | 4948 | OR MD 1.23 (1.03 - 1.96) |  |  |  |
|  | Pathirana 2021 | SBP | Subgroup | > 10 years | NP | 4941 | OR MD 2.58 (1.05 - 4.11) |  |  |  |
| HDP | Sukmanee 2022 | Hypertension | Overall |  | 37 | 1517583 | RR 3.46 (2.67 - 4.49) | 99 | The funnel plot was assymetrical indicating publication bias | High |
| Pre-eclampsia with severe features | Sukmanee 2022 | Hypertension | Overall |  | 4 | 751128 | RR 6.67 (1.51 - 29.4) | 91 |  |  |
| Pre-eclampsia without severe features | Sukmanee 2022 | Hypertension | Overall |  | 2 | 770868 | RR 7.21 (0 - 17565.27) | 97 |  |  |
| Early-onset preeclampsia | Sukmanee 2022 | Hypertension | Overall |  | 2 | 858 | RR 2.53 (1.93 - 3.32) | 0 |  |  |
|  | **Subgroup analysis for individual follow-up duration (Sukmanee 2022)** | | | | | | | |  |  |
| HDP | Sukmanee 2022 | Hypertension | Subgroup | <=5 years | 7 | 321971 | RR 5.34 (2.74 - 4.49) | 98 |  |  |
|  | Sukmanee 2022 | Hypertension | Subgroup | 6-10 years | 6 | 285947 | RR 4.22 (2.19 - 8.10) | 98 |  |  |
|  | Sukmanee 2022 | Hypertension | Subgroup | 11-15 years | 7 | 786479 | RR 3.27 (2.02 - 5.30) | 97 |  |  |
|  | Sukmanee 2022 | Hypertension | Subgroup | >15 years | 2 | 745778 | RR 1.79 (1.22 – 2.61) | 0 |  |  |
|  | Sukmanee 2022 | Hypertension | Subgroup | Unspecified | 15 |  | RR 3.10 (1.81 - 5.29) | 99 |  |  |
|  | Xu 2022 | Chronic Hypertension | Overall |  | 11 | 228317 | OR 3.61 (2.18 - 6.00) | 96 | HDP: P=0.4639, PE: 0.5380 | High |
| Gestational hypertension | Xu 2022 | Chronic Hypertension | Overall |  | 3 | 933 | OR 6.24 (1.73 - 22.55) | 73 |  |  |
| Pre-eclampsia | Xu 2022 | Chronic Hypertension | Overall |  | 13 | 341060 | OR 3.19 (1.52 - 6.7) | 97 |  |  |
|  | **Subgroup analysis by region (Xu 2022)** | | | | | | | |  |  |
| HDP | Xu 2022 | Chronic Hypertension | Subgroup | North and South America | 5 | 6581 | OR 2.11 (1.42 - 3.14) | 82 |  |  |
|  | Xu 2022 | Chronic Hypertension | Subgroup | Europe | 3 | 1260 | OR 4.26 (1.05 - 17.21) | 88 |  |  |
|  | Xu 2022 | Chronic Hypertension | Subgroup | Asia | 3 | 220476 | OR 5.52 (3.01 - 10.14) | 64 |  |  |
|  | **Subgroup analysis by publication years (Xu 2022)** | | | | | | | |  |  |
|  | Xu 2022 | Chronic Hypertension | Subgroup | < 2016 | 3 | 4906 | OR 1.78 (1.04 - 3.04) | 83 |  |  |
|  | Xu 2022 | Chronic Hypertension | Subgroup | >=2016 | 8 | 223411 | OR 4.33 (2.62 - 7.16) | 91 |  |  |
|  | **Subgroup analysis by study design (Xu 2022)** | | | | | | | |  |  |
|  | Xu 2022 | Chronic Hypertension | Subgroup | Cross-sectional | 1 | 768 | OR 1.21 (0.90 - 1.64) | NA |  |  |
|  | Xu 2022 | Chronic Hypertension | Subgroup | Case-control | 5 | 4712 | OR 2.47 (1.47 - 4.13) | 57 |  |  |
|  | Xu 2022 | Chronic Hypertension | Subgroup | Cohort study | 5 | 222837 | OR 5.19 (2.99 - 9.01) | 92 |  |  |
|  | **Subgroup analysis by NOS score (Xu 2022)** | | | | | | | |  |  |
|  | Xu 2022 | Chronic Hypertension | Subgroup | NOS < 7 | 8 | 224094 | OR 3.68 (2.03 - 6.68) | 96 |  |  |
|  | Xu 2022 | Chronic Hypertension | Subgroup | NOS >=7 | 3 | 4223 | or 3.21 (1.19 - 8.66) | 39 |  |  |
|  | **Subgroup analysis by sample size (Xu 2022)** | | | | | | | |  |  |
|  | Xu 2022 | Chronic Hypertension | Subgroup | Sample size < 500 | 6 | 228317 | OR 4.26 (1.94 - 9.33) | 69 |  |  |
|  | Xu 2022 | Chronic Hypertension | Subgroup | Sample size >= 500 | 5 | 227217 | OR 3.21 (1.62 - 6.35) | 98 |  |  |
| Pre-eclampsia | **Subgroup analysis by region (Xu 2022)** | | | | | | | |  |  |
|  | Xu 2022 | Chronic Hypertension | Subgroup | Asia | 3 | 6529 | OR 7.54 (2.49 - 22.81) | 36 |  |  |
|  | Xu 2022 | Chronic Hypertension | Subgroup | Europe | 4 | 1582 | OR 2.19 (0.30 - 16.02) | 98 |  |  |
|  | Xu 2022 | Chronic Hypertension | Subgroup | North and South America | 6 | 332949 | OR 3.32 (1.26 - 8.74) | 95 |  |  |
|  | **Subgroup analysis by publication years (Xu 2022)** | | | | | | | |  |  |
|  | Xu 2022 | Chronic Hypertension | Subgroup | < 2016 | 5 | 1830 | OR 1.54 (0.28 - 8.44) |  |  |  |
|  | Xu 2022 | Chronic Hypertension | Subgroup | >=2016 | 8 | 339230 | OR 5.53 (3.21 - 9.53) |  |  |  |
|  | **Subgroup analysis by study design (Xu 2022)** | | | | | | | |  |  |
|  | Xu 2022 | Chronic Hypertension | Subgroup | Case-control | 5 | 332188 | OR 2.68 (0.45 - 15.86) | 90 |  |  |
|  | Xu 2022 | Chronic Hypertension | Subgroup | Cohort study | 8 | 8872 | OR 3.70 (1.22 - 11.22) | 98 |  |  |
|  | **Subgroup analysis by NOS score (Xu 2022)** | | | | | | | |  |  |
|  | Xu 2022 | Chronic Hypertension | Subgroup | NOS < 7 | 5 | 331949 | OR 6.88 (6.07 - 7.80) | 0 |  |  |
|  | Xu 2022 | Chronic Hypertension | Subgroup | NOS >=7 | 8 | 9111 | OR 2.15 (0.70 - 6.64) | 98 |  |  |
|  | **Subgroup analysis by sample size (Xu 2022)** | | | | | | | |  |  |
|  | Xu 2022 | Chronic Hypertension | Subgroup | Sample size < 500 | 8 | 699 | OR 4.05 (1.12 - 14.69) | 75 |  |  |
|  | Xu 2022 | Chronic Hypertension | Subgroup | Sample size >= 500 | 5 | 340361 | OR 2.69 (0.97 - 7.45) | 99 |  |  |

GDM: Gestational diabetes mellitus, HDP – hypertensive disorders of pregnancy, SBP – Systolic Blood Pressure, DBP – Diastolic Blood Pressure, T2DM – Type 2 diabetes mellitus

## **FigS2:** meta-analysis of the primary studies that excluded chronic hypertension from the three systematic reviews


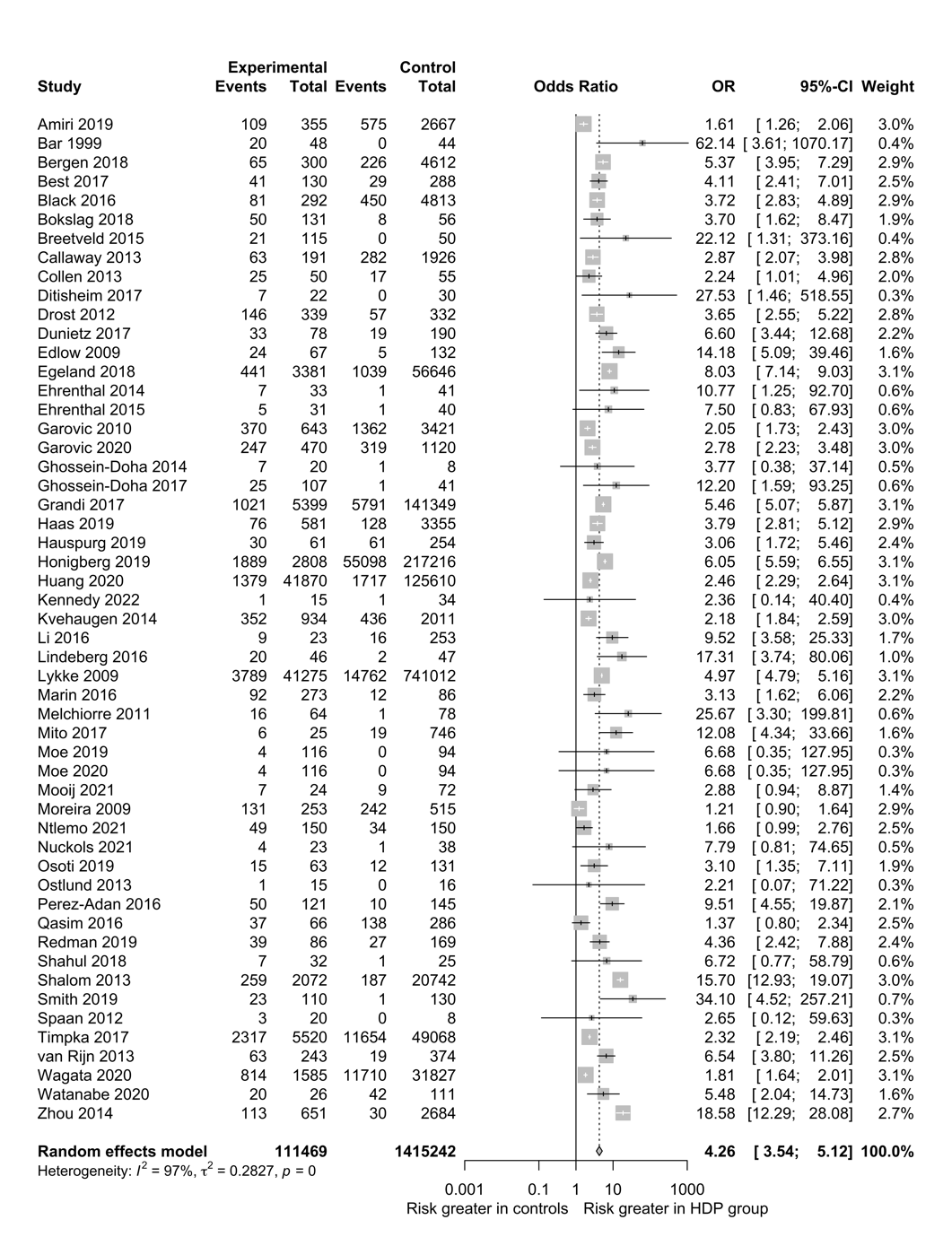


## Table S11: PRIOR Checklist

PRIOR Checklist

(Gates M, Gates A, Pieper D, et al. Reporting guideline for overviews of reviews of healthcare interventions: development of the PRIOR statement. *BMJ* 2022;378:e070849. doi:10.1136/bmj-2022-070849.)

| Section Topic | # | Item | Location reported |
| --- | --- | --- | --- |
| TITLE | |  |  |
| Title | 1 | Identify the report as an overview of reviews. | Line 2 |
| ABSTRACT | |  |  |
| Abstract | 2 | Provide a comprehensive and accurate summary of the purpose, methods, and results of the overview of reviews. | Line 33 to 78 |
| INTRODUCTION | |  |  |
| Rationale | 3 | Describe the rationale for conducting the overview of reviews in the context of existing knowledge. | Line 85 to 106 |
| Objectives | 4 | Provide an explicit statement of the objective(s) or question(s) addressed by the overview of reviews. | Line 108 to 110 |
| METHODS | |  |  |
| Eligibility criteria | 5a | Specify the inclusion and exclusion criteria for the overview of reviews. If supplemental primary studies were included, this should be stated, with a rationale. | Line 129 to 134 |
|  | 5b | Specify the definition of ‘systematic review’ as used in the inclusion criteria for the overview of reviews. | Line 129 to 134 |
| Information sources | 6 | Specify all databases, registers, websites, organizations, reference lists, and other sources searched or consulted to identify systematic reviews and supplemental primary studies (if included).  Specify the date when each source was last searched or consulted. | Line 136 to 137 |
| Search strategy | 7 | Present the full search strategies for all databases, registers and websites, such that they could be reproduced. Describe any search filters and limits applied. | Table 2 |
| Selection process | 8a | Describe the methods used to decide whether a systematic review or supplemental primary study (if included) met the inclusion criteria of the overview of reviews. | Line 148 to 150 |
|  | 8b | Describe how overlap in the populations, interventions, comparators, and/or outcomes of systematic reviews was identified and managed during study selection. | Line 180 to 198 |
| Data collection process | 9a | Describe the methods used to collect data from reports. | Line 152 to 154 |
|  | 9b | If applicable, describe the methods used to identify and manage primary study overlap at the level of the comparison and outcome during data collection. For each outcome, specify the method used to illustrate and/or quantify the degree of primary study overlap across systematic reviews. | Line 180 to 198 |
|  | 9c | If applicable, specify the methods used to manage discrepant data across systematic reviews during data collection. | Line 152 to 154 |
| Data items | 10 | List and define all variables and outcomes for which data were sought. Describe any assumptions made and/or measures taken to identify and clarify missing or unclear information. | Line 119 to 126  Line 208 to 209 |
| Risk of bias assessment | 11a | Describe the methods used to *assess* risk of bias or methodological quality of the included systematic reviews. | Line 161 to 178 |
|  | 11b | Describe the methods used to *collect* data on (from the systematic reviews) and/or *assess* the risk of bias of the primary studies included in the systematic reviews. Provide a justification for instances where flawed, incomplete, or missing assessments are identified but not re-assessed. | Line 161 to 178 |
|  | 11c | Describe the methods used to *assess* the risk of bias of supplemental primary studies (if included). | Nott applicable |
| Synthesis methods | 12a | Describe the methods used to summarize or synthesize results and provide a rationale for the choice(s). | Line 200 to 222 |
|  | 12b | Describe any methods used to explore possible causes of heterogeneity among results. | Line 213 to 215 |
|  | 12c | Describe any sensitivity analyses conducted to assess the robustness of the synthesized results. | NA |
| Reporting bias assessment | 13 | Describe the methods used to *collect* data on (from the systematic reviews) and/or *assess* the risk of bias due to missing results in a summary or synthesis (arising from reporting biases at the levels of the systematic reviews, primary studies, and supplemental primary studies, if included). | Line 160 to 178 |
| Certainty assessment | 14 | Describe the methods used to *collect* data on (from the systematic reviews) and/or *assess* certainty (or confidence) in the body of evidence for an outcome. | Line 213 to 217 |
| RESULTS | |  |  |
| Systematic review and supplemental primary study selection | 15a | Describe the results of the search and selection process, including the number of records screened, assessed for eligibility, and included in the overview of reviews, ideally with a flow diagram. | Figure 1 |
|  | 15b | Provide a list of studies that might appear to meet the inclusion criteria, but were excluded, with the main reason for exclusion. | Supplementary materials Table 3 |

| Section Topic | # | Item | Location reported |
| --- | --- | --- | --- |
| Characteristics of systematic reviews and supplemental primary studies | 16 | Cite each included systematic review and supplemental primary study (if included) and present its characteristics. | Table 4 |
| Primary study overlap | 17 | Describe the extent of primary study overlap across the included systematic reviews. | Line 241 to 263  Supplementary Table 5a to 5g |
| Risk of bias in systematic reviews, primary studies, and supplemental primary studies | 18a | Present assessments of risk of bias or methodological quality for each included systematic review. | Line 234 to 240  Supplementary Table 4 |
|  | 18b | Present assessments (*collected* from systematic reviews or *assessed* anew) of the risk of bias of the primary studies included in the systematic reviews. | Supplementary Table 4 |
|  | 18c | Present assessments of the risk of bias of supplemental primary studies (if included). | NA |
| Summary or synthesis of results | 19a | For all outcomes, summarize the evidence from the systematic reviews and supplemental primary studies (if included). If meta-analyses were done, present for each the summary estimate and its precision and measures of statistical heterogeneity. If comparing groups, describe the direction of the effect. | Table 5 |
|  | 19b | If meta-analyses were done, present results of all investigations of possible causes of heterogeneity. | Table 5 and Supplementary Figure 1 |
|  | 19c | If meta-analyses were done, present results of all sensitivity analyses conducted to assess the robustness of synthesized results. | NA |
| Reporting biases | 20 | Present assessments (*collected* from systematic reviews and/or *assessed* anew) of the risk of bias due to missing primary studies, analyses, or results in a summary or synthesis (arising from reporting biases at the levels of the systematic reviews, primary studies, and supplemental primary studies, if included) for each summary or synthesis assessed. | Figure 2 and  Supplementary Table 4 |
| Certainty of evidence | 21 | Present assessments (*collected* or *assessed* anew) of certainty (or confidence) in the body of evidence for each outcome. | Table 5 and Figure 2 and strengths and limitations |
| DISCUSSION | | |  |
| Discussion | 22a | Summarize the main findings, including any discrepancies in findings across the included systematic reviews and supplemental primary studies (if included). | Line 509 to 525 |
|  | 22b | Provide a general interpretation of the results in the context of other evidence. | Line 555 to 565 |
|  | 22c | Discuss any limitations of the evidence from systematic reviews, their primary studies, and supplemental primary studies (if included) included in the overview of reviews. Discuss any limitations of the overview of reviews methods used. | Line 526 to 544 |
|  | 22d | Discuss implications for practice, policy, and future research (both systematic reviews and primary research). Consider the relevance of the findings to the end users of the overview of reviews, e.g., healthcare providers, policymakers, patients, among others. | Line 579 to 603 |
| OTHER INFORMATION | | |  |
| Registration and protocol | 23a | Provide registration information for the overview of reviews, including register name and registration number, or state that the overview of reviews was not registered. | Line 116 to 118 |
|  | 23b | Indicate where the overview of reviews protocol can be accessed, or state that a protocol was not prepared. | Line 116 to 118 |
|  | 23c | Describe and explain any amendments to information provided at registration or in the protocol. Indicate the stage of the overview of reviews at which amendments were made. | NA |
| Support | 24 | Describe sources of financial or non-financial support for the overview of reviews, and the role of the funders or sponsors in the overview of reviews. | Line 621 to 635 |
| Competing  interests | 25 | Declare any competing interests of the overview of reviews' authors. | Line 619 to 620 |
| Author information | 26a | Provide contact information for the corresponding author. | Line 25 to 27 |
|  | 26b | Describe the contributions of individual authors and identify the guarantor of the overview of reviews. | Line 612 to 618 |
| Availability of data and other materials | 27 | Report which of the following are available, where they can be found, and under which conditions they may be accessed: template data collection forms; data collected from included systematic reviews and supplemental primary studies; analytic code; any other materials used in the overview of reviews. | Supplementary materials and Table 5 |
